# Precision and bias of spatial capture–recapture estimates: A multi‐site, multi‐year Utah black bear case study

**DOI:** 10.1002/eap.2618

**Published:** 2022-05-17

**Authors:** Greta M. Schmidt, Tabitha A. Graves, Jordan C. Pederson, Sarah L. Carroll

**Affiliations:** ^1^ Department of Biology San Diego State University San Diego California USA; ^2^ U.S. Geological Survey, Northern Rocky Mountain Science Center West Glacier Montana USA; ^3^ Utah Division of Wildlife Resources Kamas Utah USA; ^4^ Graduate Degree Program in Ecology Colorado State University Fort Collins Colorado USA

**Keywords:** abundance, bias, density, detection, hierarchical models, home range, oSCR, population ecology, precision, spatial capture–recapture, uncertainty, *Ursus americanus*

## Abstract

Spatial capture–recapture (SCR) models are powerful analytical tools that have become the standard for estimating abundance and density of wild animal populations. When sampling populations to implement SCR, the number of unique individuals detected, total recaptures, and unique spatial relocations can be highly variable. These sample sizes influence the precision and accuracy of model parameter estimates. Testing the performance of SCR models with sparse empirical data sets typical of low‐density, wide‐ranging species can inform the threshold at which a more integrated modeling approach with additional data sources or additional years of monitoring may be required to achieve reliable, precise parameter estimates. Using a multi‐site, multi‐year Utah black bear (*Ursus americanus*) capture–recapture data set, we evaluated factors influencing the uncertainty of SCR structural parameter estimates, specifically density, detection, and the spatial scale parameter, sigma. We also provided some of the first SCR density estimates for Utah black bear populations, which ranged from 3.85 to 74.33 bears/100 km^2^. Increasing total detections decreased the uncertainty of density estimates, whereas an increasing number of total recaptures and individuals with recaptures decreased the uncertainty of detection and sigma estimates, respectively. In most cases, multiple years of data were required for precise density estimates (<0.2 coefficient of variation [CV]). Across study areas there was an average decline in CV of 0.07 with the addition of another year of data. One sampled population with very high estimated bear density had an atypically low number of spatial recaptures relative to total recaptures, apparently inflating density estimates. A complementary simulation study used to assess estimate bias suggested that when <30% of recaptured individuals were spatially recaptured, density estimates were unreliable and ranged widely, in some cases to >3 times the simulated density. Additional research could evaluate these requirements for other density scenarios. Large numbers of individuals detected, numbers of spatial recaptures, and precision alone may not be sufficient indicators of parameter estimate reliability. We provide an evaluation of simple summary statistics of capture–recapture data sets that can provide an early signal of the need to alter sampling design or collect auxiliary data before model implementation to improve estimate precision and accuracy.

## INTRODUCTION

Estimating abundance and density of wildlife populations with accuracy and precision is fundamental to ecological research and has critical implications for effective wildlife conservation and management efforts. Over the past 15 years, spatial capture–recapture (SCR) models have emerged, advanced, and become the standard for robust population abundance and density estimation, particularly for wide‐ranging species that violate the assumptions of geographic closure (Borchers & Efford, 2008; Royle & Young, 2008). SCR models update strictly temporal capture–recapture frameworks by using spatiotemporal encounter histories of unique individuals to account for spatial variation in detection probability across the landscape. They model detection as a function of trap locations relative to the estimated center point of an individual's space use, known as its activity center (Royle, Chandler, Sollmann, et al., 2013). The highest probability of detecting an animal is at its activity center, with declining detection probability as distance from the activity center increases. The area in which density is estimated is a discretized plane referred to as the state space, which encompasses the trap array and extends outward to the surrounding area to include all activity centers available for detection. This allows for an explicit estimation of density and, thus, population size (Gardner et al., 2010). Covariates can be attributed to the state space, and density can be modeled as a function of these spatial attributes (Sutherland et al., 2019). Ultimately, SCR models have proven to be a rich and flexible model framework with which to derive precise population estimates and to understand more fully the drivers of spatial variation in abundance and density (Fuller et al., 2016; Kendall et al., 2019; Murphy et al., 2019; Royle, Chandler, Gazenksi, et al., 2013; Royle, Chandler, Sun, et al., 2013).

Parameter estimation using SCR requires obtaining adequate sample sizes of unique individuals, recaptures, and spatial recaptures (Royle, Chandler, Sollmann, et al., 2013). The number of individuals detected, coupled with the estimated detection probability within the state space, informs the density estimates (Efford & Boulanger, 2019). The most commonly used detection function, the half‐normal model, comprises two parameters that are informed by recaptures and spatial recaptures. First, detections and recaptures of unique individuals across sampling occasions inform the baseline detection probability estimates at the activity center. Second, spatial recaptures supply distance measurements between traps and latent animal activity centers that allow for estimation of the spatial parameter that defines individuals' space use and controls the rate of decline of the detection function as the distance from the activity center increases (Efford, 2004; Royle & Young, 2008).

To effectively inform wildlife conservation and management decision‐making, SCR parameter estimates must be reliable, meeting the criteria of both precision and accuracy (Paterson et al., 2019). Given that sufficient data are required for parameter estimation, precision and accuracy of estimates are expected to be influenced by sample sizes. For SCR, sample size has multiple measures. The number of individuals, recaptures, and spatial recaptures are each relevant for the hierarchical model. For example, we expect precision and accuracy to increase for density estimates as the number of individuals detected increases (Morin et al., 2018), for baseline detection probability estimates as the number of total detections and recaptures increases (Wilton et al., 2014), and for sigma estimates as the number of spatial recaptures increases (Morin et al., 2018).

Elusive, wide‐ranging, or depleted carnivore populations are characterized by low densities and low detection rates (Linden et al., 2017; Wilton et al., 2014). Even given an ideal study design, sampling these populations often results in sparse data sets with few unique detections and limited spatial recaptures (Howe et al., 2013). This is especially relevant to genetic capture–recapture sampling in remote areas. This technique is commonly used to sample carnivore populations but, given the logistic challenges and common budget constraints, it often results in a limited number of sampling sites or revisits and correspondingly sparse data sets. Whereas simulation‐focused studies have provided a baseline for understanding the performance of SCR models given a range of sampling scenarios (Clark, 2019; Paterson et al., 2019; Sollmann et al., 2012; Sun et al., 2014), assessment of the consequences of low sample sizes on SCR parameter estimate uncertainty in empirical data sets has been limited. Implementation of SCR modeling across real‐world capture–recapture data sets with a range of sample sizes can serve to evaluate when SCR is an effective tool for deriving precise parameter estimates versus when detection rates and population densities are too low for the model framework to perform well. In these cases, a more integrated modeling approach with additional data sources or additional years of monitoring may be required to improve parameter estimator precision and coverage (Morin et al., 2018; Paterson et al., 2019; Royle, Chandler, Sun, et al., 2013; Sollmann et al., 2013).

From 2004 to 2011, the Utah Division of Wildlife Resources (UDWR) sampled five black bear (*Ursus americanus*) populations distributed across mountainous regions in the State using the same hair snare design (Pederson et al., 2012). Each study area represented a population of management interest, encompassing a spectrum of population characteristics. The data sets covered a range of sample sizes varying by the number of individuals detected, recaptures, and spatial recaptures. The data sets also varied across study areas in properties derived from sample sizes, including the ratio of individuals with spatial recaptures to all recaptured individuals and average recaptures per detected individual. Additionally, although a few studies (Morehouse & Boyce, 2016; Morin et al., 2018) have used multiple sampling years to bolster sample sizes by sharing parameters across years, assessment of the effectiveness of this practice as a strategy to increase estimate precision has been limited. These long‐term empirical data sets provide a valuable opportunity to test the performance of SCR when estimating population parameters of a wide‐ranging, low‐density carnivore species.

Therefore, to evaluate how capture–recapture data set characteristics influence structural parameter estimate uncertainty, we applied the SCR model framework to this archived multi‐site, multi‐year black bear genetic capture–recapture data set. To address both the precision and accuracy of SCR estimates and explore the mechanisms driving underlying patterns in our empirical data, we also conducted a complementary simulation study, creating data sets with similar characteristics to our black bear capture–recapture data. Finally, we evaluated the differences among study areas in best‐supported models, describing variation in density, detection, and the spatial scale parameter sigma, providing insight about variation among populations of Utah black bears. A better understanding of how SCR models perform with limited, but realistic, sample sizes will allow researchers to more effectively employ SCR techniques to derive informative estimates of the population parameters crucial to ecological research and management.

## METHODS

### Study area and archived data

**FIGURE 1:**
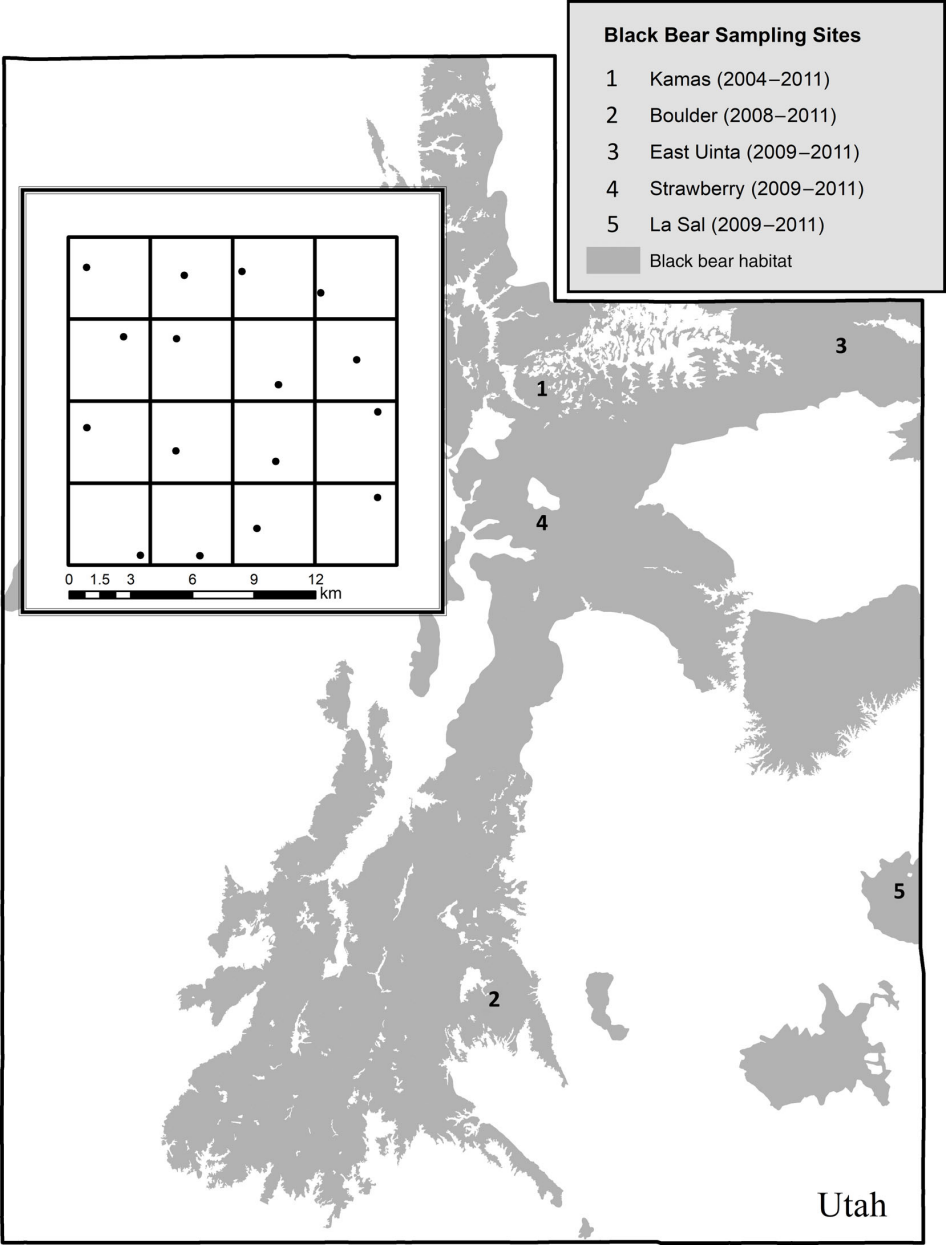
Locations of the five Utah study areas selected to sample black bear populations. Inset shows the Kamas sampling array, which is representative of the 16 km × 16 km grid sampling scheme used at each of the study areas, where a hair snare collection corral (black dot) was established in each grid cell

Our analysis incorporated five distinct study areas (Kamas, Boulder, Strawberry, East Uinta, La Sal) located in mountainous regions across the eastern portion of Utah, each sampled for 3–6 years within 2004–2011 (Figure 1 and Table 1). At each site, researchers established a 256 km^2^ sampling grid separated into 4 km × 4 km cells, totaling 16 cells per site. The only exception to this sampling design occurred at the Kamas study area in 2004, where the 256 km^2^ grid was sampled more intensively with 3.2 × 3.2 km cells, resulting in 25 sampling cells. Each cell had a hair collection corral constructed with barbed wire and baited with a scent lure as described in Pederson et al. (2012). Beginning in June each year, researchers visited each cell every 14 days over four sampling occasions. On each sampling occasion, all potential black bear hair samples were collected, labeled, and dried. To assign an individual identity and sex to each hair sample, Wildlife Genetics International (Nelson, British Columbia, Canada) or Dr. Karen Mock with Utah State University (Logan, Utah, USA) used six microsatellite markers and an additional amelogenin marker as detailed in Pederson et al. (2012).

**TABLE 1:** Available years of sampling data for each study area, with individuals detected, estimated density, estimated baseline detection, estimated sigma, total detections, total recaptures, and total spatial recaptures for single‐year models

Site	Year	*n*	D (bears/100 km^2^)	*p*_0_	σ (km)	Detections	Recaptures	Spatial recaptures
Kamas	2004	13	2.32 (0.36)	0.12 (0.28)	4.19 (0.19)	35	22	17
	2005	15	4.54 (0.31)	0.23 (0.35)	2.59 (0.18)	28	13	7
	2006	14	4.71 (0.34)	0.15 (0.51)	2.76 (0.2)	24	10	6
	2007	17	5 (0.28)	0.24 (0.33)	2.65 (0.14)	34	17	8
	2008	20	4.66 (0.34)	0.11 (0.34)	3.95 (0.21)	37	17	10
	2011	20	3.19 (0.32)	0.16 (0.31)	4.68 (0.18)	40	20	11
Boulder	2008	13	5.32 (0.38)	0.12 (0.51)	2.59 (0.26)	21	8	5
	2009	19	8.96 (0.36)	0.13 (0.52)	2.43 (0.22)	26	7	5
	2011	17	5.82 (0.31)	0.26 (0.34)	2.88 (0.17)	32	15	10
East Uinta	2010	17	10.44 (0.42)	0.18 (0.51)	1.66 (0.22)	23	6	1
	2011	16	8.73 (0.44)	0.1 (0.57)	2.23 (0.24)	21	5	3
Strawberry	2009	14	5.58 (0.36)	0.1 (0.43)	3.01 (0.23)	24	10	7
	2010	18	4.68 (0.44)	0.07 (0.37)	4.48 (0.29)	33	15	9
	2011	7	3.03 (0.5)	0.01 (1.58)	2.79 (0.24)	13	6	3
La Sal	2009	62	58.05 (0.25)	0.15 (0.31)	1.5 (0.14)	77	15	5
	2010	58	44.33 (0.24)	0.11 (0.32)	1.97 (0.14)	74	16	7
	2011	74	36.94 (0.17)	0.14 (0.22)	2.38 (0.11)	110	36	17

*Note*: Parameter estimates are from precision comparison models. Coefficient of variation (standard error of estimate/estimate, coefficient of variation [CV]) for parameter estimates are shown in parentheses, 0.20 CV is generally indicative of a reasonably precise estimate (Pollock et al., 1990).

### SCR model

To fit SCR models, we used the package *oSCR* (Sutherland et al., 2019), which uses maximum likelihood estimation, in R version 3.6.3 (R Core Team, 2020). We formatted the individually identified black bear capture–recapture data from each study area into multi‐session (3–6 years) capture histories that retained spatial information on individual $\left(i\right)$ detections at hair collection corrals $\left(j=16,25\right)$ across sampling occasions $\left(k=4\right)$, such that *y*_ijk_ ∼ Bernoulli(*p*_ij_). The detection model component defines probability of detection of an individual at a particular trap (*p*_ij_) as a function of distance from the individual's activity center $s_{i}$ to that trap having location $x_{j}$. We used the half‐normal model:

pij=p0×exp−distxjsi22σ2,

where $p_{0}$ is the baseline encounter probability, and σ is the spatial scale parameter determining the rate of decrease in detection probability in regard to the distance between $x_{j}$ and $s_{i}$.

To ensure that density estimates were insensitive to the designation of the state space size, we fitted null models for all study areas, testing a range of buffer sizes around the hair collection locations (5–20 km). For each study area, we buffered the sampling array by the distance at which density estimates stabilized and removed non‐habitat (e.g., reservoirs) from the state spaces if necessary. We set a conservatively fine state space resolution of 0.25 km^2^ pixel size based on the recommendation that resolution should be less than half the expected estimate of sigma (Sutherland et al., 2019).

### Ecological model selection

To estimate density for each study area and assess factors influencing density and detection, we used an Akaike information criterion (AIC)‐based model selection process for each study area (Burnham & Anderson, 2004). We considered individual and trap‐level variation in detection and sigma, landscape‐level variation in density, and year‐specific variation in all parameters. We used all years of data available for each study area in a multi‐session model and applied a sequential model selection approach, first identifying the most parsimonious detection model, then using that detection model to evaluate year‐specific and landscape‐level variation in density for each study area (Kendall et al., 2009, 2019; Sutherland et al., 2018). At each model selection step, we used AIC to select best‐supported models within 2 ΔAIC of the top model that included only informative parameters (i.e., 85% confidence interval did not cover 0; Arnold, 2010; Sutherland et al., 2018; Wisdom et al., 2020).

For the detection model, we first tested for effects of sex, year, and a sex–year interaction on the sigma parameter. We then used the best‐supported covariates for sigma to build a full detection model. We assessed four potential influences on baseline detection probability. We considered a trap covariate quantifying average percentage canopy cover within a 270 m^2^ area around each trap using the 2011 United States Forest Service Tree Canopy Analytical Percent Cover layer (Coulston et al., 2012). We evaluated a scent covariate indexing the rank effect of scent lure applied at a hair snare corral that we binned into four categories representing increasing attraction, as in Pederson et al. (2012). We assessed a trap‐specific behavioral covariate to model trap‐happy or trap‐shy behavioral response after first detection. Finally, we tested for year‐specific variation in detection.

Using the best‐supported detection model, we fitted models evaluating percentage canopy cover (Coulston et al., 2012), elevation (USGS, 2019), and year‐specific density as three candidate covariates on the density parameter. We aggregated percentage cover and elevation layers to a 500 m × 500 m cell size to match the resolution of the state space. Covariates were centered by subtracting each covariate layer by its mean and then scaled by dividing centered layers by their standard deviations using the scale() function in R before model implementation. In addition to reporting the best‐supported model for each study area (Table 2), a full list of models were evaluated, see Appendix S1: Tables S1–S3.

**TABLE 2:** Ecological model selection results of best‐supported models for each study area with coefficients and associated standard errors for density, baseline detection probability, and sigma

Study area	Model	Covariate	Coefficient	Estimate	SE
Kamas	Density	Intercept	*D*_intercept_	−4.64	0.15
	Detection	Intercept	*p*_0_	−2.22	0.20
		Behavior	*p*_behavior_	1.10	0.28
		Trap canopy cover	*p*_trap canopy cover_	0.51	0.10
	Sigma	Intercept	sig_intercept_	8.34	0.10
Boulder	Density	Intercept	*D*_intercept_	−3.82	0.25
		Elevation	*D*_elevation_	−0.70	0.27
	Detection	Intercept	*p*_0_	−1.59	0.28
	Sigma	Intercept	sig_intercept_	7.97	0.14
East Uinta	Density	Intercept	*D*_intercept_	−3.24	0.39
		Canopy cover	*D*_canopy cover_	−1.79	0.43
	Detection	Intercept	*p*_0_	−1.96	0.42
		Trap canopy cover	*p*_trap canopy cover_	0.97	0.29
	Sigma	Intercept	sig_intercept_	7.19	0.24
		Year	sig_year2_	0.44	0.20
			sig_year3_	0.47	0.20
Strawberry	Density	Intercept	*D*_intercept_	−4.50	0.24
	Detection	Intercept	*p*_0_	−2.48	0.28
		Trap canopy cover	*n*_trap canopy cover_	0.84	0.17
	Sigma	Intercept	sig_intercept_	8.18	0.16
La Sal	Density	Intercept	*D*_intercept_	−1.60	0.24
		Elevation	*D*_elevation_	−0.33	0.15
	Detection	Intercept	*p*_0_	−2.18	0.39
		Behavior	*p*_behavior_	1.32	0.35
		Trap canopy cover	*p*_trap canopy cover_	0.20	0.08
		Scent	*p*_scent_	−0.17	0.08
	Sigma	Intercept	sig_intercept_	7.73	0.13
		Year	sig_year2_	0.00	0.09
			sig_year3_	0.20	0.09

*Note*: Covariates include percentage canopy cover (canopy cover), elevation, trap‐specific behavioral covariate (behavior), canopy cover around the hair collection site (trap canopy cover) ranked scent category used at a trap (scent), and year‐specific variation (year). Tables containing all models considered in our sequential model selection approach with ΔAIC and model weights can be found in Appendix S1: Tables S1–S3. SE, standard error.

### Evaluating precision with empirical data sets

To evaluate factors related to SCR estimate precision, we fitted SCR models to estimate density, detection, and the spatial scale parameter sigma for a single year, partial combinations of years, and the full number of years available for each of the five black bear population study areas. For example, for a study area sampled from 2009 to 2011, in addition to fitting models using only data from each year (2009, 2010, and 2011) we also ran models that used 2 and 3 years of data (2009 + 2010, 2009 + 2011, 2010 + 2011, and 2009 + 2010 + 2011). We used a simple detection model using covariates that shared support across study areas in the study area‐specific best‐supported detection models from the ecological model selection process detailed above and in Appendix S1: Tables S1–S3. In multi‐year models, we specified density, detection, and the scale parameter sigma to be shared across all years, which increased the sample size for estimation of the shared parameters and provided a single standard error estimate for each model parameter that we could relate to data set sample sizes.

We considered two metrics describing maximum likelihood estimate precision: standard error (SE) and the coefficient of variation (CV; SE_Estimate_/Estimate). When back‐transforming model parameters from the link scale to the original scale, the oSCR package calculates SE using the delta method by taking the square root of the Hessian of the likelihood function evaluated at the maximum likelihood estimates (Sutherland et al., 2019). In addition to SE, we quantified precision as the CV of the structural parameter estimates for density, detection, and sigma (Efford & Boulanger, 2019). We focused primarily on the CV, as it is a standardized measure of dispersion around the estimate and therefore more suitable for cross‐population comparisons when there are large differences in estimated population densities (Walther & Moore, 2005). Lower CV values indicate greater precision, and we considered precise estimates to be those with a CV < 0.2 (Efford & Boulanger, 2019; Pollock et al., 1990). However, Efford and Boulanger (2019) noted that this threshold is only sufficient if managers are interested in detecting large changes in population size (>64% decrease, >96% increase). If detecting finer scale changes in populations is of interest, lower CVs will be needed. We reported both SE and CV because our aim was to explore any identifiable patterns related to precision and uncertainty of parameter estimates.

### Relating data set characteristics to precision metrics

To understand how summarized attributes of the sampling data are related to the uncertainty of SCR structural parameter estimates, we evaluated a suite of capture–recapture data set characteristics (Table 3). We assessed sample sizes that either have been shown or are expected to influence the uncertainty of parameter estimates including the total number of unique individuals, detections, recaptures, and spatial recaptures (Efford & Boulanger, 2019; Morin et al., 2018). We also included the sample sizes of unique individuals with recaptures and unique individuals with spatial recaptures. Additionally, we considered capture–recapture data set characteristics derived from sample sizes, such as the ratio of individuals with spatial recaptures to individuals recaptured only at the same trap. We also evaluated the average number of detections, recaptures, and spatial recaptures per individual as potential variables associated with estimate precision.

**TABLE 3:** Comparison of candidate models hypothesized to influence precision, quantified as either standard error (SE) or coefficient of variation (CV), for spatial capture–recapture (SCR) structural parameter estimates density, baseline detection probability, and sigma

Candidate Model		CV		SE	
Parameter	Data Attribute	ΔAIC_c_	*w*_*i*_	ΔAIC_c_	*w*_*i*_
Density~	Detections	0	1	174.77	0
	Recaptures	49.41	0	136.47	0
	Unique individuals	82.55	0	166.93	0
	Spatial recaptures	95.96	0	107.29	0
	Recaptures to detections	211.63	0	19.37	0
	Average detections per individual	212.1	0	25.33	0
	Average recaptures per individual	213.03	0	0	1
	Average spatial recaptures per individual	216.04	0	28.04	0
	Spatial recaptures to detections	216.85	0	54.86	0
Detection~	Recaptures	0	0.76	6.93	0.03
	Individuals with recaptures	2.35	0.24	12.13	0
	Individuals with spatial recaptures	16.94	0	0	0.95
	Detections	28.61	0	7.72	0.02
	Unique individuals	95.42	0	31.16	0
	Average detections per individual	165.18	0	74.56	0
	Individuals with spatial recaptures to detections	185.5	0	79.46	0
	Individuals with spatial recaptures to individuals with recaptures	189.05	0	70.39	0
Sigma~	Individuals with recaptures	0	1	10.82	0
	Detections	20.1	0	0	1
	Recaptures	55.42	0	31.17	0
	Individuals with spatial recaptures	70.95	0	33.24	0
	Spatial recaptures	100.32	0	41.69	0
	Average spatial recaptures per individual	213.12	0	52.34	0
	Spatial recaptures to recaptures	221.17	0	41.6	0
	Individuals with spatial recaptures to individuals with recaptures	221.25	0	23.3	0

The spatial scale parameter sigma is an essential component of the detection model that describes the rate of decay in detection probability from an individual's activity center. The distribution of distances between detections at traps and the estimated activity centers of recaptured individuals inform the sigma parameter in the detection component of the model. When individuals are detected in multiple traps (i.e., spatial recaptures), this provides information for estimating sigma. Because longer distance spatial recaptures are inherently rarer than short‐distance spatial recaptures, they have a large influence on the tail of the distribution (Nathan et al., 2003). Further, where an individual is not detected also informs the sigma estimate by decreasing the detection probability at that trap, at the distance between the estimated activity center and the trap. When individuals are detected multiple times at the same trap (i.e., simple recaptures) and not at additional traps, the distance to the activity center is likely to be small. The ratio of short‐distance or same‐trap recaptures to long‐distance recaptures might therefore influence the precision of the resulting sigma estimate. Thus, to evaluate the influence of the number of spatial recaptures on estimate uncertainty, we calculated the ratio of spatial recaptures to total recaptures and the ratio of individuals with spatial recaptures relative to the total number of individuals with recaptures, which includes individuals with spatial recaptures and simple recaptures.

To assess how these capture–recapture data set characteristics covaried with SE and CV associated with the three SCR structural parameter estimates (density, detection, and sigma), we used generalized linear models with a gamma distribution and dispersion set to one to hold variance constant across the range of the covariates. Because many sample size characteristics of the capture–recapture data sets were highly correlated, we only considered explanatory variables in a univariate context. For each of the three structural parameter estimates we evaluated candidate models based on AIC_c_ and considered top‐ranked models to be representative of the sampling data attributes most associated with the selected metric (SE, CV) quantifying estimate precision.

To directly evaluate change in the CV of density as a function of incorporating additional years of sampling data, we fitted a linear regression with the number of years of data as a covariate. In the regression we evaluated models with 1–3 years of data, as Kamas was the only study area with more than 3 years of available sampling data.

### Assessing bias through simulations

We examined density estimates for signals of inaccuracy (i.e., appreciably lower or higher than expected) by comparing SCR density estimates with previous records of black bear density in the sampling regions specifically (Frost, 1990) and the arid mountains of the Southwest generally (Gould et al., 2018). We conducted exploratory simulations to further assess the driving factors and consequences of identified concerns with sampling data attributes (Appendix S2: Section S1). We simulated capture–recapture data on a 16 km × 16 km state space that matched the 16‐trap array used in each black bear study area. Adapting code from Sutherland et al. (2019), we simulated capture histories at three densities (2, 20, and 60 bears/100 km^2^), encompassing the range of density estimates from the five sampled populations (Schmidt, 2021: R code to run SCR simulations.R). We fixed sigma at 2 km, which is on the lower range of sigma estimates from the empirical data sets and is within the range of acceptable sigma values given trap spacing (Sollmann et al., 2012; Sun et al., 2014). We simulated capture histories for each density with probability of detection at the activity center, *p*_0_, set at 0.15. Additionally, we simulated a second set of capture histories at each density that included a trap‐specific behavioral covariate on detection, setting initial *p*_0_ at 0.07 and detection probability after first detection (*p*_*b*_) at 0.15, based on the detection parameters estimated at the La Sal study area with a trap‐specific behavioral covariate on detection (Appendix S1: Figure S5). We simulated a total of six different scenarios with three densities with and without a behavioral effect for each density. We simulated 500 capture histories for each scenario. For the three scenarios without a behavioral effect, we fitted the null model. For the three scenarios simulated with a behavioral effect, we fitted the null model and a model with trap‐specific behavioral covariate on detection. All models were fitted using a conservatively large 17 km buffer and fine 0.25 km^2^ state space resolution. We evaluated bias and confidence interval coverage of density estimates for the nine different models arising from the six density simulation scenarios.

## RESULTS

### Ecological model selection

In preliminary model development to determine an ideal state space buffer size, density estimates stabilized above 10 km, so we conservatively used a 15 km buffer around each of our study areas. For each of the five study areas, comprehensive model selection results are included in Appendix S1: Tables S1–S3, and we reported the best full model and associated coefficient estimates for each study area in Table 2. The best‐supported models for density varied across study areas. For the Kamas and Strawberry study areas, the best‐supported model suggested homogenous density. The Kamas study area had an estimated density of 3.85 bears/100 km^2^ (95% CI = 2.85–5.18), and the Strawberry study area had an estimated density of 4.43 bears/100 km^2^ (95% CI = 2.76–7.12). Increasing elevation was associated with a decrease in density at the Boulder (average density of 9.06 bears/100 km^2^, 95% CI = 4.65–17.98) and La Sal (average density of 74.33 bears/100 km^2^, 95% CI = 46.22–128.30) study areas. Density decreased with increasing canopy cover at the East Uinta study area (average density of 35.80 bears/100 km^2^, 95% CI = 12.64–103.52). The La Sal and East Uinta study areas were among the largest black bear density estimates recorded in the western USA (Gould et al., 2018), whereas the other sites fell within a more typical range for black bear populations in arid habitats (Baldwin & Bender, 2012; Gould et al., 2018). Variation in density across years was not supported at any study area.

Top detection models also varied across study areas (Table 2; Appendix S1: Table S2), although the covariate quantifying canopy cover around each trap had support across four of the five areas. Baseline detection probability estimates ranged from 0.08 to 0.17 and sigma estimates ranged from 1.33 to 4.18 km across all sites, which are low but fall within expected ranges based on previous black bear SCR research (Clark, 2019; Gardner et al., 2010; Wilton et al., 2014). Year‐specific variation in sigma was supported at the East Uinta and La Sal study areas.

### Evaluating precision with empirical data sets

**FIGURE 2:**
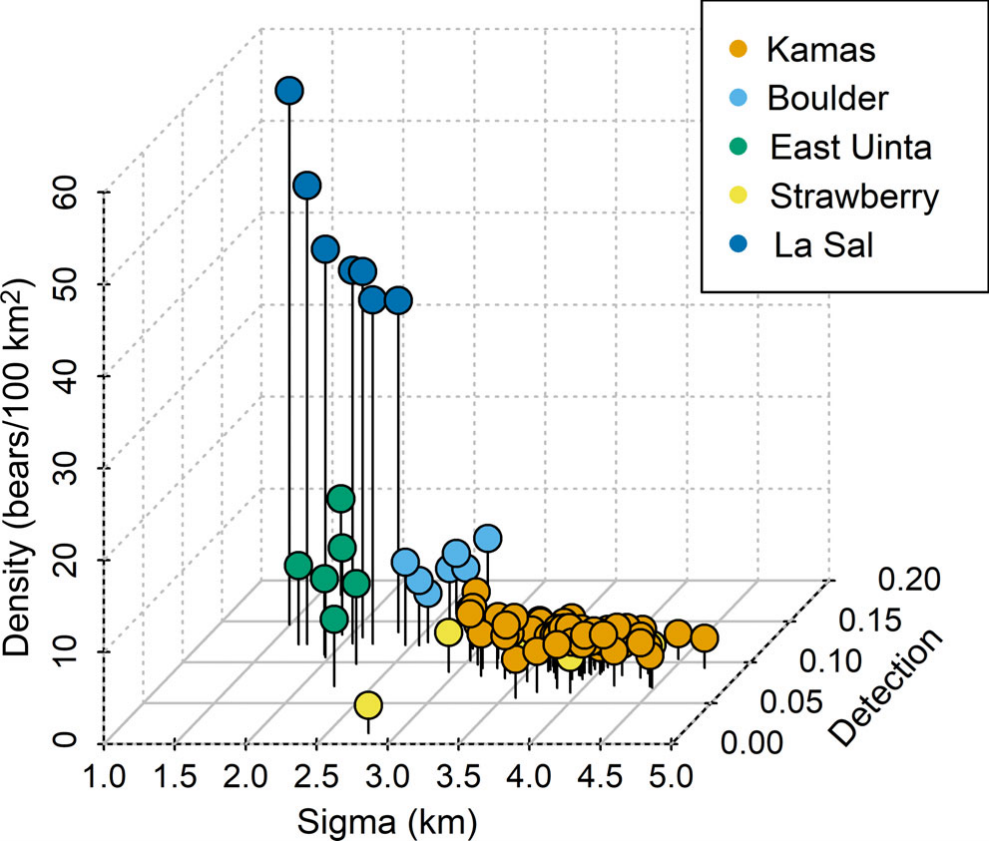
Estimates of density, baseline detection probability, and sigma at each black bear study area (Boulder, East Uinta, Kamas, La Sal, Strawberry) for single, partial, and full multi‐year combinations of precision comparison models (*n* = 90)

Using all possible combinations of sampling years available for each site, we fitted models to seven combinations of years of data (e.g., 2009, 2010, 2011, 2009 + 2010, 2010 + 2011, 2009 + 2011, and all years) for sites with 3 years of data (Boulder, East Uinta, Strawberry, and La Sal) and 63 combinations of years of data for the 6 years of data at the Kamas site. We used canopy cover as a covariate on baseline detection probability. After eliminating the 2009 model from the East Uinta site, which did not converge, across all sites and combinations of sampling years we had 90 estimates of density, baseline detection probability, and sigma with associated sampling data characteristics and measures of uncertainty (Figure 2; Schmidt, 2021; CR_summaries_estimates.csv). The number of individuals detected in a single year ranged from 6 to 74. Density estimates ranged from 2.32 to 58.05 bears/100 km^2^ across all sites. Baseline detection probability estimates ranged from 0.01 to 0.26 and sigma estimates ranged from 1.50 to 4.68 km across all study areas.

### Relating data set characteristics to precision metrics

**FIGURE 3:**
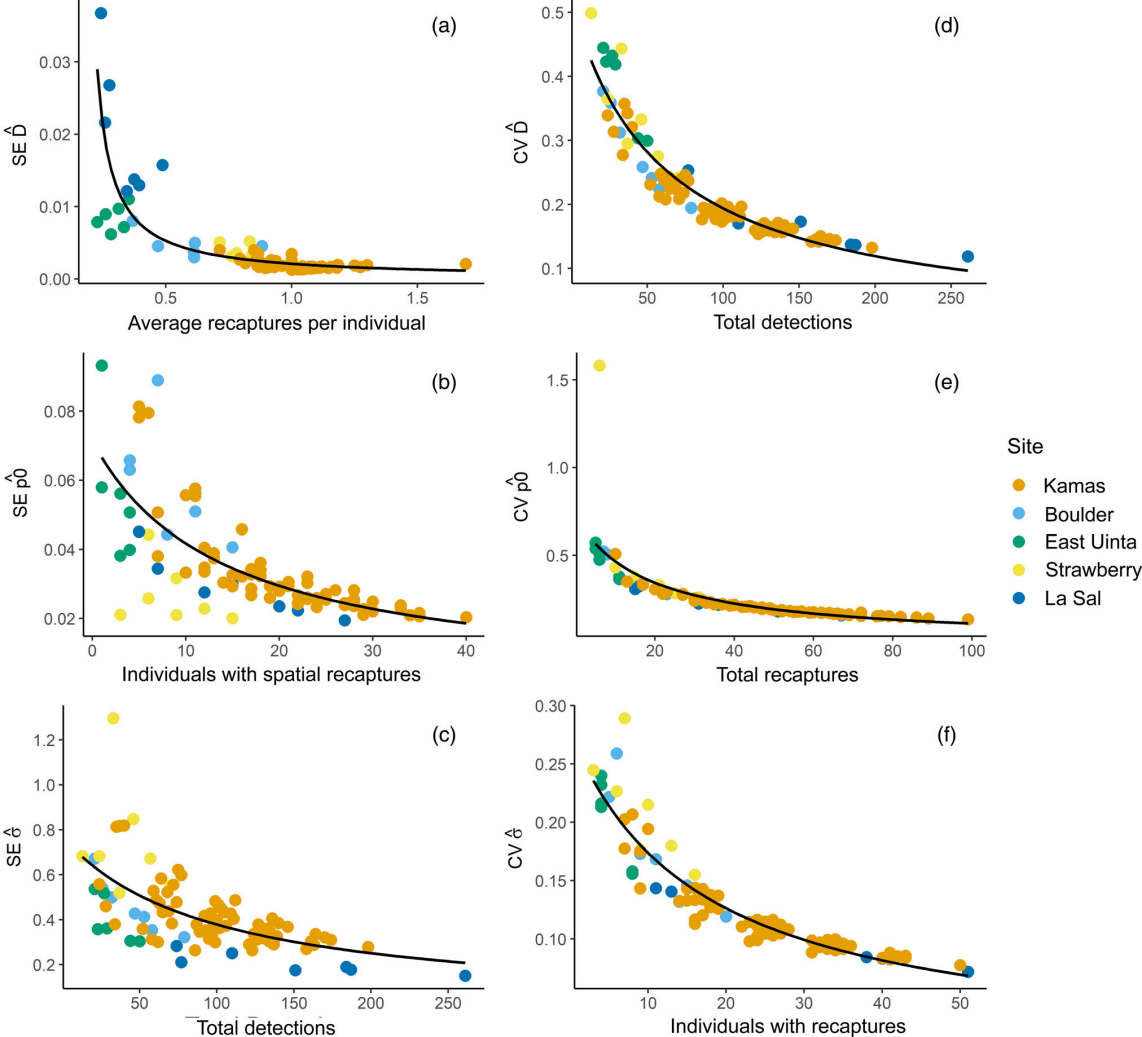
Best‐supported models of capture–recapture data sample sizes associated with structural estimate precision showing (a) standard error (SE) of the density estimate as a function of average recaptures per individual, (b) SE of the baseline detection estimate as a function of individuals with spatial recaptures, (c) SE of the sigma estimate as a function of total detections, (d) coefficient of variation (CV) of the density estimate as a function of total detections, (e) CV of the baseline detection estimate as a function of total recaptures, and (f) CV of the sigma estimate as a function of the number of individuals with recaptures

When considering SE as the metric of precision, best‐supported models indicated that increasing the average number of recaptures per individual decreased the SE of density (Figure 3a), increasing the number of individuals with spatial recaptures decreased the SE of detection (Figure 3b), and increasing the total detections decreased the SE of sigma (Figure 3c and Table 3; Appendix S3: Figure S3).

When considering CV as the primary metric of precision, the best‐supported model for precision of the density estimate suggested that increasing detections best explained increased precision (Figure 3d). Increasing precision of the baseline detection probability parameter was best explained by increasing the number of recaptures (Figure 3e). The best‐supported model for precision of the sigma estimate, the spatial scale parameter of the detection model, suggested a positive relationship between the number of individuals with recaptures and sigma precision (Figure 3f).

**FIGURE 4:**
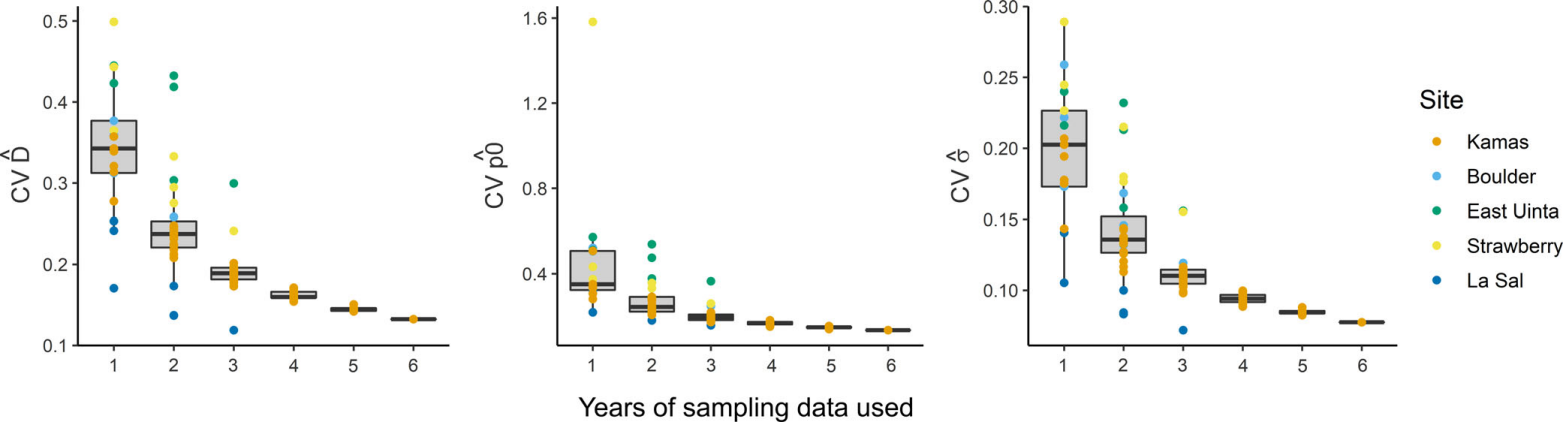
Coefficient of variation (CV) for estimates of density (left), baseline detection probability (middle), and sigma (right), across 1–6 years of sampling data. Note that sampling for >3 years only occurred at the Kamas study area

As models included additional years of sampling data (1–3 years), uncertainty around the density estimate decreased (β = −0.07, 95% CI = −0.06 to −0.09, *p* < 0.001), as additional years increased the total number of detections and recaptures available to inform estimates (Table 1). Visual assessment suggests that, with more than 3 years of sampling data, which existed only for the Kamas study area, the decrease in precision conferred by adding years may attenuate (Figure 4). Across the five study areas, only one of the density estimates (La Sal, 2011) using 1 year of data met the precision standard of <0.2 CV. Sampling at the La Sal study area yielded more detected individuals than the other sampled sites, which resulted in more total detections to inform SCR structural parameter estimates (average of 87 detections and 22 recaptures per sampling year). For two of the remaining study areas, Kamas (average of 33 detections and 17 recaptures per sampling year) and Boulder (average of 26 detections and 10 recaptures per sampling year), single‐year models were insufficiently precise; however, an additional 2 years of data decreased uncertainty of estimates such that estimates met the 0.2 CV threshold. Strawberry (average of 23 detections and 10 recaptures per sampling year) and East Uinta (average of 22 detections and six recaptures per sampling year) study areas both had very sparse single‐year data sets that for some years failed to converge. For these study areas, multi‐year models using 3 years of available data improved the precision of estimates but did not increase data input sample sizes enough to decrease CV below 0.2 (Figure 4; Schmidt, 2021; CR_summaries_estimates.csv). For baseline detection estimates, Strawberry and Boulder study areas were sufficiently precise with 1 year of data, whereas the La Sal study area required 2 years of data, and the Kamas study area required 3 years of data. The East Uinta study area detection estimates did not meet the precision threshold even with models using all 3 years of data available. Four out of five study areas met the 0.2 CV precision threshold for sigma estimates with just 1 year of data, although the East Uinta study area needed 3 years of data for a sufficiently precise sigma estimate.

### Assessing bias through simulations

Density estimates at the La Sal study area (36.94–58.05 bears/100 km^2^ in precision comparison models, 74.33 bears/100 km^2^ in best ecological model) were substantially larger than densities reported 20 years prior (13 bears/100 km^2^; Frost, 1990). Frost (1990) reported ad hoc calculations based on intensive trapping in the region rather than capture–recapture estimates, but provided the only reported density for this study area prior to this research. Our SCR density estimates for the La Sal study area were among the highest reported for black bear populations in the West (Gould et al., 2018; Mace & Chilton‐Radandt, 2011; Stetz et al., 2014, 2019; Welfelt et al., 2019). Long‐term harvest data from this region suggested increasing harvests (10 permits and one harvest in the La Sal Unit in 1990, 50 permits and 27 harvests in the La Sal Unit in 2011; Bernales & DeBloois, 2018), which is usually interpreted as indicative of increasing abundance (Wolfe et al., 2016). However, our SCR point estimates for density suggested very large declines in the abundance of 129–215 bears in a single year, which is unusual (Table 1). These large fluctuations in abundance suggested inaccurate results that appeared unrelated to factors associated with estimate precision, given that La Sal had the most precise estimates among our precision comparison models. A trap‐specific behavioral term was strongly supported at the La Sal study area in the ecological model selection (Table 2; Appendix S1: Table S2). A behavioral term in the detection model increased density estimates relative to models without a behavioral term, but also increased confidence intervals such that coverage of the true density increased (best ecological model: 95% CI = 46.22–128.30; precision comparison model: 95% CI = 31.32–50.35).

**FIGURE 5:**
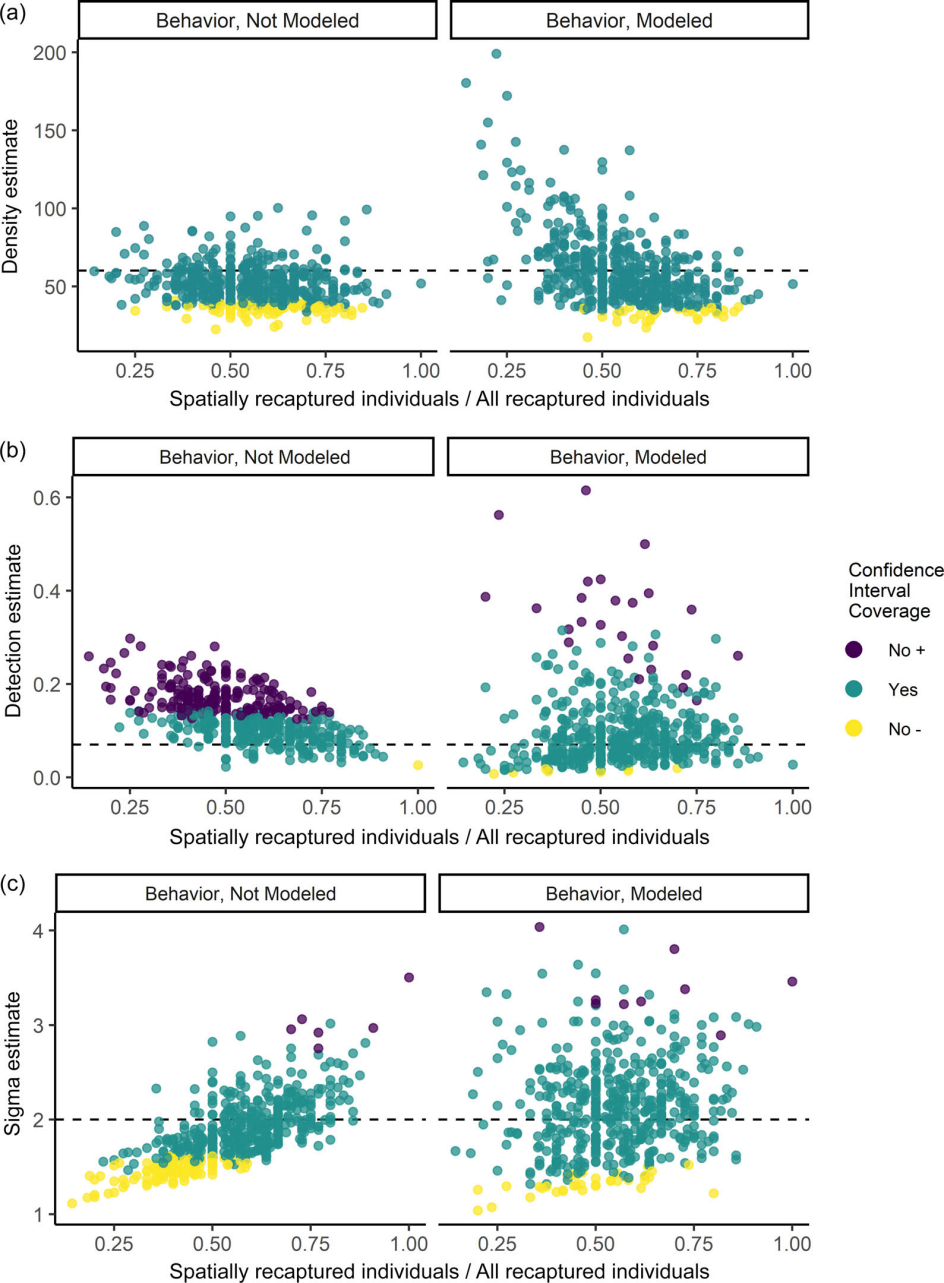
Ratio of spatially recaptured individuals to all recaptured individuals (i.e., spatial recaptured + recaptured only at trap of first detection) from simulated data sets with a trap‐happy behavioral effect on detection and associated point estimates for (a) density (bears/100 km^2^), (b) detection and (c) sigma (km), with the true simulated parameter value indicated by the dashed black line. Capture–recapture data sets were simulated at 60 bears/100 km^2^, with a baseline detection of 0.07, with a trap‐specific behavioral effect increasing to 0.15 after first detection. We fitted models that did (left column) and did not (right column) include the behavioral term. Sigma was set at 2 km. Color of the estimate indicates that confidence interval coverage for that parameter was below the true value (yellow), encompassed the true value (green–gray), or was above the true value (dark purple)

In general, our simulations supported the same relationships with precision of SCR parameter estimates and attributes of the sampling data identified in our empirical data sets (Appendix S2: Figure S4). For the especially low simulated density of 2 bears/100 km^2^, very few animals were detected or recaptured, leading to issues with convergence, estimate precision, and some bias (Appendix S2: Tables S1–S5). These issues were most closely reflected in the East Uinta and Strawberry study areas, which also had low amounts of capture–recapture information. Simulations with a positive trap‐specific behavioral term and simulated densities comparable with the La Sal site (60 bears/100 km^2^) included cases with approximately the same low ratios of spatially recaptured individuals to all recaptured individuals that we observed in the real data, although these simulated cases generally had lower sample sizes of spatial recaptures than the empirical data from the La Sal study area (Appendix S2: Figure S1). At this density (60 bears/100 km^2^), data simulated with a trap‐specific behavioral effect leading to a low ratio of spatially recaptured individuals to total recaptured individuals (≤0.3, 5% of simulations, *n* = 22) produced density point estimates that were biased high ($\bar{x}$ = 109.97 ± 43.68 bears/100 km^2^) relative to estimates above this threshold (>0.3) of the ratio of spatially recaptured individuals to total recaptured individuals ($\bar{x}$ = 57.71 ± 18.60 bears/100 km^2^). However, estimates had large confidence intervals that covered the true simulated value despite inaccurate and mostly high point estimates (Figure 5). Density and detection estimates had no to low bias for simulation scenarios with densities of 20 and 60 bears/100 km^2^ given the ratios of spatially recaptured individuals to total recaptured individuals >0.3 and a model specified to match the simulation (i.e., no behavior simulation fit with a no behavior model, behavior effect simulation fit with a behavior model; Appendix S2: Table S3). Sigma estimates were unbiased under the same conditions, except that at 60 bears/100 km^2^ and no behavioral effect, sigma estimates had a consistent positive bias, although as noted above density and detection estimates were unbiased in this scenario (Appendix S2: Figure S2).

## DISCUSSION

Implementing capture–recapture analyses can be a costly, time‐consuming, and computationally intensive process, and our research provides simple data set summary statistics that provide researchers with early indications of issues related to estimate uncertainty and inaccuracy that can inform more effective model implementation. When sampling wildlife populations that are difficult to observe, the data collected are rarely plentiful. In a case in which density estimates were higher than expected, we identified that a very low proportion of individuals with spatial recaptures relative to total recaptured individuals may be a potential signal suggesting when inflated estimates could occur.

We further identified attributes related to the quantity and quality of input data that influenced SCR model performance. As expected, we found that with a low number of individuals detected, few recaptures, and few spatial recaptures, models either failed to converge or produced estimates that did not meet the objective for the standard of precision (≤0.2 CV) in a single‐year context. Variation in population patterns across study areas suggests that model performance and precision can be increased, and bias mitigated, when SCR sampling and model implementation strategies are tailored to each sampled population. Even for populations of the same species in one geographic region, ecological differences among habitats and population dynamics can influence the ability to collect sufficient data. Our simulations demonstrated that the role of spatial recaptures may be particularly important for reducing bias and inflated estimates, even when density and the number of detected individuals is relatively high. Additionally, differences among best‐supported full models evaluating landscape heterogeneity in density among study areas indicated that multiple study sites capturing different ranges of landscape variability could reveal conditions in which landscape attributes drove population processes, better informing larger scale management objectives (Short Bull et al., 2011). These insights provided additional tools when applying the SCR framework to wildlife populations of conservation and management concern.

For density estimates across study areas, associated precision was sensitive to the total number of detections, consistent with SCR simulation evaluations (Paterson et al., 2019). The total number of recaptures was the best‐supported attribute of the sampling data describing variation in detection estimate precision, and the number of individuals with recaptures was the best‐supported attribute associated with sigma estimate precision. Although density is the primary parameter of interest to ecologists, increased precision in the detection model parameter estimates (i.e., base detection probability, sigma) can ensure more reliable SCR density estimates (Murphy et al., 2019). This information may also be useful for studies in which wildlife connectivity, interactions with humans, or the interactions of space use and density are of interest (Efford et al., 2016).

Adding multiple years of data to inform SCR estimates increased all capture–recapture data set sample sizes considered and, in turn, increased the precision of the three structural parameter estimates, although beyond 3 years of data this effect appeared to wane (Figure 4). For models used to compare estimate precision, we modeled density as a shared parameter across years, so that we could evaluate the influence of sampling data attributes on precision for the parameters of density, detection, and sigma. However, the best‐supported ecological models of some study areas did include year‐specific variation (Table 2), and in many cases year‐specific parameter estimates, especially density, may be of primary interest (Morin et al., 2018). When this is the objective a subset of parameters, such as the detection parameters, could be shared among years, which is also expected to increase precision relative to modeling years separately (Appendix 1: Figure S5, Howe et al., 2013), but the overall decrease in the CV may be less.

Individual and spatial heterogeneity are important considerations for SCR estimate reliability and utility. The top models from each study area supported some level of heterogeneity in the density, detection, or sigma parameters, underscoring the importance of obtaining sufficient sample sizes to effectively test for spatial, temporal, and individual variation in the population and observational processes in the SCR model. Previous research evaluating landscape attributes related to gene flow in Mountain West black bear populations has shown that considering multiple study areas that capture greater variability in landscape features can reveal the conditions under which some features are supported as influential to population processes (Short Bull et al., 2011). Even among study areas with similar habitats, higher variability within a study area could explain why a habitat feature is supported as influential in one study area but not another. This concept is crucial for managers interested in applying SCR models describing landscape heterogeneity in density from one study area to a larger scale, for example state‐wide management planning.

The best full SCR model for the Kamas study area (Appendix S1: Table S3) did not include the same covariates supported in the CR models of Pederson et al. (2012). It is possible that variation in detection described by ranked scent attractant and sex in the CR models were better explained in the SCR framework by the variation in detection due to the spatial placement of traps relative to individual activity centers, or by the other spatial covariates we tested such as canopy cover around the trap site. Previous research has shown that unmodeled heterogeneity in the spatial and observational process models causes negative bias in density estimates (Gerber & Parmenter, 2015; Howe et al., 2013; Tobler et al., 2015). This could explain the lower estimated population size at the Kamas study area in Pederson et al. (2012), which ranged from 15 (95% CI = 12–20) to 22 (95% CI = 19–26) estimated individuals across a 4‐year time span relative to SCR point estimates of 40 (95% CI = 20–81) to 87 (95% CI = 50–150) individuals across the same time span. Importantly, the CR and SCR estimates are not directly comparable, because SCR refers to the number of activity centers within the state space, whereas CR estimates refer to the superpopulation in an undefined area (Kendall, 1999).

Sampling years at the La Sal study area when fewer individuals were detected and there was a low ratio of spatially recaptured individuals to total recaptured individuals yielded higher density and lower sigma estimates (Table 1). We observed a similar pattern at the East Uinta study area, although the scant data from the East Uinta sampling years created more obvious issues with model convergence in precision comparison models and unreliable estimates in the best‐supported ecological model (average density of 35.80 bears/100 km^2^, 95% CI = 12.64–103.52). Ecological model selection supported a trap‐specific behavioral effect on the detection process at the La Sal study area. In this more fully specified model, density estimates increased relative to models lacking a behavioral effect and confidence intervals increased, suggesting that this further reduced point estimate accuracy. Our simulations further supported the idea that in some populations with a behavioral response, overly large density estimates can occur when the ratio of individuals with spatial recaptures to all individuals with recaptures is low (<0.3), potentially reducing the mean distance among recaptures and the activity center and constraining sigma estimates. Therefore, we suspect that the La Sal estimates with the trap behavior are biased high.

Observational or ecological processes could be responsible for individuals caught repeatedly at one trap without any spatial recaptures. Large numbers of recaptures at the same trap can occur when there is a “trap‐happy” behavioral response to a sampling site. At the La Sal study area, this behavior could have been a response to scent attractant placed at the hair collection corrals, or the activity range could be constrained by hunting and pursuit with dogs that is prevalent at this study area during the spring, summer, and fall. In some instances, this behavioral response could temporarily reduce the size of the activity range and therefore inflate density estimates.

In the distance sampling literature, best practices exist to account for sampling biases using both left and right truncation of distance data (Alldredge & Gates, 1985; Borchers et al., 2006; Howe et al., 2017), and left truncation can be used to reduce both negative and positive bias (Beaver et al., 2014; Ruette et al., 2003). Right truncation of samples at large distances has been used in SCR modeling when movements far exceeded those representative of the general population (Kendall et al., 2019). Left truncation of spatial recapture distances has not been explored in the SCR literature, but could be a useful method to account for positive bias in the detection model incurred by the sampling data. For example, if individuals have an excessive number of repeated recaptures at one trap across multiple sampling occasions, it may be useful to assess the sensitivity of density to removal of one or more same‐site recaptures. However, if the number of spatial recaptures is simply too low, truncation may not be an appropriate method to resolve bias. Our empirical results and simulations suggested that more than three spatial recaptures were needed for models to converge and to avoid gross overestimations of density. If there are very few spatial recaptures, incorporating additional data sources to inform space use and the detection model, such as telemetry data (Royle, Chandler, Sun, et al., 2013; Sutherland et al., 2019), may be the only way to address this issue.

Our results build on previous studies exploring the performance of SCR models, adding to current best practices in the SCR data collection and analysis workflow (Table 4). Trap spacing of 2σ or smaller and a trapping extent greater than one individual home range are basic requirements for avoiding biased estimates and ensuring adequate sample sizes of detections and spatial recaptures for model convergence (Clark, 2019; Sollmann et al., 2012; Sun et al., 2014). Additionally, a clustered sampling approach can cover greater spatial extents to sample more individuals, while still allowing for spatial recaptures to estimate sigma (Clark, 2019). This strategy would probably be most effective at increasing the number of short‐distance spatial recaptures, and therefore best suited to higher density populations with small home ranges. Baited traps can be used in SCR sampling designs (Humm et al., 2017; Molina et al., 2017; Pederson et al., 2012; Welfelt et al., 2019), but the work presented here suggests that, although this strategy can increase detections, it may result in unreliable estimates if spatial recaptures do not also increase relative to same‐trap detections. Additional traps that are close to each other may help to address this issue. A transect or search‐encounter sampling design in which recaptures at the same location are limited could be beneficial for avoiding a behavioral response to trap arrays, if feasible. However, Efford (2019) cautioned that this sampling design could incur strong bias if animal home ranges are uniformly elongated due to two‐dimensional habitat features. Efford and Boulanger (2019) proposed that an evaluation of expected sample sizes of individuals and recaptures using simulations can be related to expected precision, which can be used to inform optimal SCR sampling design. Similarly, Dupont et al. (2021) further identified a genetic algorithm that optimized an objective function related to estimator precision to more quickly identify an optimal design based on a goal of maximizing the number of individuals detected, the number of spatial recaptures, or a balance of these two criteria. Finally, multiple study areas can be used to better capture variability in landscape features when landscape heterogeneity in density is being evaluated or when inference across a larger landscape is of interest (Short Bull et al., 2011).

**TABLE 4:** A workflow of SCR best practices

Issue	Suggested approach	Reference
Study design		
Effective trap array design	Trap spacing <2σ, trapping extent >one home range, consider clustered approach, moving traps between sampling sessions, transect design, implications of using bait and whether a behavioral response is expected to occur	Clark (2019), Sollmann et al. (2012), Sun et al. (2014), This study
Require precise estimates	Evaluate expected precision based on expected parameter estimates to optimize design	Dupont et al. (2021), Efford and Boulanger (2019)
Low sample size expected	Provision for additional data sources including telemetry data and additional sampling sessions, or consider multiple detection methods	Howe et al. (2013), Morehouse and Boyce (2016), Paterson et al. (2019), Welfelt et al. (2019)
Evaluating influence of landscape heterogeneity on density and detection	Incorporate multiple sites within a management area that capture ranges of variability of landscape features of interest to determine whether patterns are present under different conditions	Short Bull et al. (2011), This study
Data evaluation		
Sufficient data for effective SCR implementation	Evaluate attributes of the sampling data, including no. detections, recaptures, and the proportion of individuals with spatial recaptures to all individuals with recaptures	Morin et al. (2018), Efford and Boulanger (2019), This study
Large spatial recapture distances affect sigma estimate	Evaluate maximum distance moved by year to look for outliers, consider left or right truncation	Kendall et al. (2019), This study
Model implementation		
Low sample size precludes convergence	Integrate additional data sources including additional sampling sessions	Howe et al. (2013), Morehouse and Boyce (2016), Royle, Chandler, Gazenksi, et al. (2013)
Potential bias incurred from spatial recaptures	Test sensitivity of estimates to left and right truncation, if unable to address, consider implications for estimate interpretation	Kendall et al. (2019), This study
Potential bias incurred from heterogeneity in detection and density	Adequately identify and model sources of heterogeneity. The ability to do this may be dependent on integrating additional data sources	Efford (2019), Howe et al. (2013)

Once SCR sampling data are collected, evaluating available sample sizes and sampling data attributes can inform model implementation and evaluation. The importance of multiple years of sampling data to increasing the precision of SCR parameter estimates in our case study reinforces the importance of identifying potential additional data. These could be in the form of multiple sampling years (Howe et al., 2013; Morehouse & Boyce, 2016; Morin et al., 2018), an increased number of sampling occasions within years (Clark, 2019), telemetry data (Royle, Chandler, Sun, et al., 2013), or other additional sources of detection data such as live trapping or harvest data (Paterson, 2019; Welfelt et al., 2019). Incorporating additional data sources can ensure that models can still be fitted and that results are sufficiently precise, even when capture–recapture data are scant or populations are small. Additional data can be both temporal and spatial, and other studies have explored sharing detection and sigma parameters across spatially separate sites to collectively improve SCR estimates (Horn et al., 2020; Howe et al., 2013; Morin et al., 2018). However, if habitat quality varies across the sites, this could potentially influence sigma (Efford et al., 2016).

Recent SCR research has increasingly targeted methods to increase the precision of model estimates (Clark, 2019; Dupont et al., 2021; Efford & Boulanger, 2019; Kristensen & Kovach, 2018; Morin et al., 2018). Our research highlights factors associated with SCR parameter estimate precision, given sparse, realistic data sets and provides approaches to increase precision and to explore potential sources of bias in the sampling data that could influence point estimate accuracy. The black bear study areas considered in our analyses were sampled with a limited, but reasonable, trap array design that yielded a range of sample sizes typical of capture–recapture studies targeting low‐density, wide‐ranging carnivore species (Gardner et al., 2010; Molina et al., 2017; Wilton et al., 2014). With our multi‐site approach, we found that, even within one geographic region where five areas were sampled using the same study design, resulting data sets varied in total detections, unique individuals, and recaptures. Additionally, resulting best‐supported ecological models varied in covariates that were influential to the observation and density components of the model. Therefore, in addition to previously identified best practices for SCR study design, we suggest collecting supplemental data, which could include additional movement data or capture–recapture sampling years to inform sparse data sets and account for potential sampling issues that may arise. Through developing a set of best practices (Table 4) spanning the complete SCR workflow of study design, data collection and evaluation, and model implementation, researchers can increase their confidence that resulting parameter estimates are accurate and sufficiently precise to provide useful information to ecologists and managers.

## CONFLICT OF INTEREST

The authors declare no conflict of interest.

## Supporting information


Appendix S1

Appendix S2

Appendix S3

## Data Availability

Data and code (Schmidt, 2021), specifically code adapted from (Sutherland et al., 2019) to run simulations, available via Figshare: https://doi.org/10.6084/m9.figshare.14368823.

## References

[eap2618-bib-0001] Alldredge, J. R. , and C. E. Gates . 1985. “Line Transect Estimators for Left‐Truncated Distributions.” Biometrics 41(1): 273–80. 10.2307/2530663. 4005381

[eap2618-bib-0002] Arnold, T. W. 2010. “Uninformative Parameters and Model Selection Using Akaike's Information Criterion.” The Journal of Wildlife Management 74(6): 1175–8. 10.2193/2009-367.

[eap2618-bib-0003] Baldwin, R. A. , and L. C. Bender . 2012. “Estimating Population Size and Density of a Low‐Density Population of Black Bears in Rocky Mountain National Park, Colorado.” European Journal of Wildlife Research 58(3): 557–66. 10.1007/s10344-011-0605-z.

[eap2618-bib-0004] Beaver, J. T. , C. A. Harper , R. E. Kissell , L. I. Muller , P. S. Basinger , M. J. Goode , F. T. V. Manen , W. Winton , and M. L. Kennedy . 2014. “Aerial Vertical‐Looking Infrared Imagery to Evaluate Bias of Distance Sampling Techniques for White‐Tailed Deer.” Wildlife Society Bulletin 38(2): 419–27. 10.1002/wsb.410.

[eap2618-bib-0005] Bernales H. , and D. DeBloois . 2018. “Utah Black Bear Annual Report 2018. Utah Department of Natural Resources, Division of Wildlife Resources.” Annual Performance Report for Federal Aid Project W‐65‐M. Segments 66 and 67.

[eap2618-bib-0006] Borchers, D. L. , and M. G. Efford . 2008. “Spatially Explicit Maximum Likelihood Methods for Capture‐Recapture Studies.” Biometrics 64(2): 377–85. 10.1111/j.1541-0420.2007.00927.x. 17970815

[eap2618-bib-0007] Borchers, D. L. , J. L. Laake , C. Southwell , and C. G. M. Paxton . 2006. “Accommodating Unmodeled Heterogeneity in Double‐Observer Distance Sampling Surveys.” Biometrics 62(2): 372–8. 10.1111/j.1541-0420.2005.00493.x. 16918901

[eap2618-bib-0008] Burnham, K. P. , and D. R. Anderson . 2004. “Multimodel Inference: Understanding AIC and BIC in Model Selection.” Sociological Methods and Research 33(2): 261–304. 10.1177/0049124104268644.

[eap2618-bib-0061] Clark, J. D. 2019. “Comparing Clustered Sampling Designs for Spatially Explicit Estimation of Population Density.” Population Ecology 61(1): 93–101. 10.1002/1438-390x.1011.

[eap2618-bib-0010] Coulston, J. W. , G. G. Moison , B. T. Wilson , M. V. Finco , W. B. Cohen , and C. K. Brewer . 2012. “Modeling Percent Tree Canopy Cover: A Pilot Study.” Photogrammetric Engineering & Remote Sensing 78(7): 715–27.

[eap2618-bib-0011] Dupont, G. , J. A. Royle , M. A. Nawaz , and C. Sutherland . 2021. “Optimal Sampling Design for Spatial Capture–Recapture.” Ecology 102(3): 1–9. 10.1002/ecy.3262. 33244753

[eap2618-bib-0012] Efford, M. 2004. “Density Estimation in Live‐Trapping Studies.” Oikos 106(3): 598–610. 10.1111/j.0030-1299.2004.13043.x.

[eap2618-bib-0013] Efford, M. G. 2019. “Non‐Circular Home Ranges and the Estimation of Population Density.” Ecology 100(2): 1–7. 10.1002/ecy.2580. 30601582

[eap2618-bib-0014] Efford, M. G. , D. K. Dawson , Y. V. Jhala , and Q. Qureshi . 2016. “Density‐Dependent Home‐Range Size Revealed by Spatially Explicit Capture–Recapture.” Ecography 39(7): 676–88. 10.1111/ecog.01511.

[eap2618-bib-0015] Efford, M. G. , and J. Boulanger . 2019. “Fast Evaluation of Study Designs for Spatially Explicit Capture–Recapture.” Methods in Ecology and Evolution 10(9): 1529–35. 10.1111/2041-210X.13239.

[eap2618-bib-0016] Frost, H. C. 1990. Population and Reproductive Characteristics of Black Bears on an Isolated Mountain in Southeastern Utah.” M.S. Thesis. Provo, UT: Brigham Young University.

[eap2618-bib-0017] Fuller, A. K. , C. S. Sutherland , J. A. Royle , and M. P. Hare . 2016. “Estimating Population Density and Connectivity of American Mink Using Spatial Capture–Recapture.” Ecological Applications 26(4): 1125–35. 10.1890/15-0315. 27509753

[eap2618-bib-0018] Gardner, B. , J. A. Royle , M. T. Wegan , R. E. Rainbolt , and P. D. Curtis . 2010. “Estimating Black Bear Density Using DNA Data from Hair Snares.” Journal of Wildlife Management 74(2): 318–25. 10.2193/2009-101.

[eap2618-bib-0019] Gerber, B. D. , and R. R. Parmenter . 2015. “Spatial Capture–Recapture Model Performance with Known Small‐Mammal Densities.” Ecological Applications 25(3): 695–705. 10.1890/14-0960.1. 26214915

[eap2618-bib-0020] Gould, M. J. , J. W. Cain , G. W. Roemer , W. R. Gould , and S. G. Liley . 2018. “Density of American Black Bears in New Mexico.” Journal of Wildlife Management 82(4): 775–88. 10.1002/jwmg.21432.

[eap2618-bib-0021] Horn, P. E. , M. J. R. Pereira , T. C. Trigo , E. Eizirik , and F. P. Tirelli . 2020. “Margay (*Leopardus wiedii*) in the Southernmost Atlantic Forest: Density and Activity Patterns under Different Levels of Anthropogenic Disturbance.” PLoS One 15(5): 1–25. 10.1371/journal.pone.0232013. PMC7202647 32374736

[eap2618-bib-0022] Howe, E. J. , S. T. Buckland , M. L. Després‐Einspenner , and H. S. Kühl . 2017. “Distance Sampling with Camera Traps.” Methods in Ecology and Evolution 8(11): 1558–65. 10.1111/2041-210X.12790.

[eap2618-bib-0023] Howe, E. J. , M. E. Obbard , and C. J. Kyle . 2013. “Combining Data from 43 Standardized Surveys to Estimate Densities of Female American Black Bears by Spatially Explicit Capture–Recapture.” Population Ecology 55(4): 595–607. 10.1007/s10144-013-0389-y.

[eap2618-bib-0024] Humm, J. M. , J. W. McCown , B. K. Scheick , and J. D. Clark . 2017. “Spatially Explicit Population Estimates for Black Bears Based on Cluster Sampling.” Journal of Wildlife Management 81(7): 1187–201. 10.1002/jwmg.21294.

[eap2618-bib-0025] Kendall, K. C. , T. A. Graves , J. A. Royle , A. C. Macleod , K. S. Mckelvey , J. Boulanger , and J. S. Waller . 2019. “Using Bear Rub Data and Spatial Capture‐Recapture Models to Estimate Trend in a Brown Bear Population.” Scientific Reports 9(16804): 1–11. 10.1038/s41598-019-52783-5. 31727927 PMC6856102

[eap2618-bib-0026] Kendall, K. C. , J. B. Stetz , J. Boulanger , A. C. Macleod , D. Paetkau , and G. C. White . 2009. “Demography and Genetic Structure of a Recovering Grizzly Bear Population.” Journal of Wildlife Management 73(1): 3–17. 10.2193/2008-330.

[eap2618-bib-0027] Kendall, W. L. 1999. “Robustness of Closed Capture–Recapture Methods to Violations of the Closure Assumption.” Ecology 80(8): 2517–25. 10.1890/0012-9658(1999)080[2517:ROCCRM]2.0.CO;2.

[eap2618-bib-0028] Kristensen, T. V. , and A. I. Kovach . 2018. “Spatially Explicit Abundance Estimation of a Rare Habitat Specialist: Implications for SECR Study Design.” Ecosphere 9(5): e02217. 10.1002/ecs2.2217.

[eap2618-bib-0029] Linden, D. W. , A. K. Fuller , J. A. Royle , and M. P. Hare . 2017. “Examining the Occupancy–Density Relationship for a Low‐Density Carnivore.” Journal of Applied Ecology 54(6): 2043–52. 10.1111/1365-2664.12883.

[eap2618-bib-0030] Mace, R. D. , and T. Chilton‐Radandt . 2011. “Black Bear Harvest Research & Management in Montana: 2011 Final Report.” Helena, MT: Montana Department of Fish, Wildlife & Parks, Wildlife Division.

[eap2618-bib-0031] Molina, S. , A. K. Fuller , D. J. Morin , and J. A. Royle . 2017. “Use of Spatial Capture–Recapture to Estimate Density of Andean Bears in Northern Ecuador.” Ursus 28(1): 117. 10.2192/ursu-d-16-00030.1.

[eap2618-bib-0032] Morehouse, A. T. , and M. S. Boyce . 2016. “Grizzly Bears without Borders: Spatially Explicit Capture–Recapture in Southwestern Alberta.” Journal of Wildlife Management 80(7): 1152–66. 10.1002/jwmg.21104.

[eap2618-bib-0033] Morin, D. J. , L. P. Waits , D. C. McNitt , and M. J. Kelly . 2018. “Efficient Single‐Survey Estimation of Carnivore Density Using Fecal DNA and Spatial Capture‐Recapture: A Bobcat Case Study.” Population Ecology 60(3): 197–209. 10.1007/s10144-018-0606-9.

[eap2618-bib-0034] Murphy, S. M. , D. T. Wilckens , B. C. Augustine , M. A. Peyton , and G. C. Harper . 2019. “Improving Estimation of Puma (*Puma concolor*) Population Density: Clustered Camera‐Trapping, Telemetry Data, and Generalized Spatial Mark‐Resight Models.” Scientific Reports 9(1): 1–13. 10.1038/s41598-019-40926-7. 30872785 PMC6418282

[eap2618-bib-0035] Nathan, R. , G. Perry , J. T. Cronin , A. E. Strand , and M. L. Cain . 2003. “Methods for Estimating Long‐Distance Dispersal.” Oikos 103(2): 261–73. 10.1034/j.1600-0706.2003.12146.x.

[eap2618-bib-0036] Paterson, J. T. , K. Proffitt , B. Jimenez , J. Rotella , and R. Garrott . 2019. “Simulation‐Based Validation of Spatial Capture‐Recapture Models: A Case Study Using Mountain Lions.” PLoS One 14(4): e0215458. 10.1371/journal.pone.0215458. PMC6474654 31002709

[eap2618-bib-0037] Pederson, J. C. , K. D. Bunnell , M. M. Conner , and C. R. McLaughlin . 2012. “A Robust‐Design Analysis to Estimate American Black Bear Population Parameters in Utah.” Ursus 23(1): 104–16. 10.2192/URSUS-D-10-00029.1.

[eap2618-bib-0038] Pollock, K. H. , J. D. Nichols , C. Brownie , and J. E. Hines . 1990. “Statistical Inference for Capture‐Recapture Experiments.” Wildlife Monographs 107: 3–97.

[eap2618-bib-0039] R Core Team . 2020. R: A Language and Environment for Statistical Computing. Vienna: R Foundation for Statistical Computing https://www.r-project.org/.

[eap2618-bib-0040] Royle, J. A. , R. B. Chandler , K. D. Gazenksi , and T. A. Graves . 2013. “Spatial Capture–Recapture Models for Jointly Estimating Population Density and Landscape Connectivity.” Ecology 94(2): 287–94. 10.1890/12-0413.1. 23691647

[eap2618-bib-0041] Royle, J. A. , R. B. Chandler , R. Sollmann , and B. Gardner . 2013. Spatial Capture‐Recapture. Cambridge, MA: Academic Press.

[eap2618-bib-0042] Royle, J. A. , R. B. Chandler , C. C. Sun , and A. K. Fuller . 2013. “Integrating Resource Selection Information with Spatial Capture‐Recapture.” Methods in Ecology and Evolution 4(6): 520–30. 10.1111/2041-210X.12205.

[eap2618-bib-0043] Royle, J. A. , and K. V. Young . 2008. “A Hierarchical Model for Spatial Capture Recapture Data.” Ecology 89(8): 2281–9. 10.1890/07-0601.1. 18724738

[eap2618-bib-0044] Ruette, S. , P. Stahl , and M. Albaret . 2003. “Applying Distance‐Sampling Methods to Spotlight Counts of Red Foxes.” Journal of Applied Ecology 40(1): 32–43. 10.1046/j.1365-2664.2003.00776.x.

[eap2618-bib-0045] Schmidt, G. 2021. “Data and Code for: Precision and Bias of Spatial Capture‐Recapture Estimates: A Multi‐Site, Multi‐Year Utah Black Bear Case Study.” Figshare, Dataset. 10.6084/m9.figshare.14368823. PMC9287071 35368131

[eap2618-bib-0046] Short Bull, R. A. , S. A. Cushman , R. MacE , T. Chilton , K. C. Kendall , E. L. Landguth , M. K. Schwartz , K. McKelvey , F. W. Allendorf , and G. Luikart . 2011. “Why Replication Is Important in Landscape Genetics: American Black Bear in the Rocky Mountains.” Molecular Ecology 20(6): 1092–107. 10.1111/j.1365-294X.2010.04944.x. 21261764

[eap2618-bib-0047] Sollmann, R. , B. Gardner , and J. L. Belant . 2012. “How Does Spatial Study Design Influence Density Estimates from Spatial Capture‐Recapture Models?” PLoS One 7(4): 1–8. 10.1371/journal.pone.0034575. PMC3335117 22539949

[eap2618-bib-0048] Sollmann, R. , B. Gardner , R. B. Chandler , D. B. Shindle , D. P. Onorato , J. A. Royle , and A. F. O'Connell . 2013. “Using Multiple Data Sources Provides Density Estimates for Endangered Florida Panther.” Journal of Applied Ecology 50(4): 961–8. 10.1111/1365-2664.12098.

[eap2618-bib-0049] Stetz, J. B. , K. C. Kendall , and A. C. Macleod . 2014. “Black Bear Density in Glacier National Park, Montana.” Wildlife Society Bulletin 38(1): 60–70. 10.1002/wsb.356.

[eap2618-bib-0050] Stetz, J. B. , M. S. Mitchell , and K. C. Kendall . 2019. “Using Spatially‐Explicit Capture–Recapture Models to Explain Variation in Seasonal Density Patterns of Sympatric Ursids.” Ecography 42(2): 237–48. 10.1111/ecog.03556.

[eap2618-bib-0051] Sun, C. C. , A. K. Fuller , and J. A. Royle . 2014. “Trap Configuration and Spacing Influences Parameter Estimates in Spatial Capture‐Recapture Models.” PLoS One 9(2): e88025. 10.1371/journal.pone.0088025. 24505361 PMC3914876

[eap2618-bib-0052] Sutherland, C. , A. K. Fuller , J. A. Royle , and S. Madden . 2018. “Large‐Scale Variation in Density of an Aquatic Ecosystem Indicator Species.” Scientific Reports 8(1): 1–10. 10.1038/s41598-018-26847-x. 29895946 PMC5997698

[eap2618-bib-0053] Sutherland, C. , J. A. Royle , and D. W. Linden . 2019. “oSCR: A Spatial Capture–Recapture R Package for Inference about Spatial Ecological Processes.” Ecography 42: 1459–69. 10.1111/ecog.04551.

[eap2618-bib-0054] Tobler, M. W. , A. Zúñiga Hartley , S. E. Carrillo‐Percastegui , and G. V. N. Powell . 2015. “Spatiotemporal Hierarchical Modelling of Species Richness and Occupancy Using Camera Trap Data.” Journal of Applied Ecology 52(2): 413–21. 10.1111/1365-2664.12399.

[eap2618-bib-0055] U.S. Geological Survey . 2019. “3D Elevation Program 1‐Meter Resolution Digital Elevation Model.” https://www.usgs.gov/core-science-systems/ngp/3dep/data-tools.

[eap2618-bib-0056] Walther, B. A. , and J. L. Moore . 2005. “The Concepts of Bias, Precision, and Accuracy, and their Use in Testing the Performance of Species Richness Estimators, with a Literature Review of Estimator Performance.” Ecography 28(July): 815–29. 10.1111/j.2005.0906-7590.04112.x.

[eap2618-bib-0057] Welfelt, L. S. , R. A. Beausoleil , and R. B. Wielgus . 2019. “Factors Associated with Black Bear Density and Implications for Management.” Journal of Wildlife Management 83(7): 1527–39. 10.1002/jwmg.21744.

[eap2618-bib-0058] Wilton, C. M. , E. E. Puckett , J. Beringer , B. Gardner , L. S. Eggert , and J. L. Belant . 2014. “Trap Array Configuration Influences Estimates and Precision of Black Bear Density and Abundance.” PLoS One 9(10): e111257. 10.1371/journal.pone.0111257. 25350557 PMC4211732

[eap2618-bib-0059] Wisdom, M. J. , R. M. Nielson , M. M. Rowland , and K. M. Proffitt . 2020. “Modeling Landscape Use for Ungulates: Forgotten Tenets of Ecology, Management, and Inference.” Frontiers in Ecology and Evolution 8(6): 1–19. 10.3389/fevo.2020.00211.

[eap2618-bib-0060] Wolfe, M. L. , E. M. Gese , P. Terletzky , D. C. Stoner , and L. M. Aubry . 2016. “Evaluation of Harvest Indices for Monitoring Cougar Survival and Abundance.” Journal of Wildlife Management 80(1): 27–36. 10.1002/jwmg.985.

