# Supplementary material for: Precision and bias of spatial capture–recapture estimates: A multi‐site, multi‐year Utah black bear case study
Source: Ecol Appl. 2022 May 17;32(5):e2618. doi: 10.1002/eap.2618 (PMC9287071; doi:10.1002/eap.2618)
Supplement: Supplementary file 1 — Appendix S1 [file EAP-32-0-s002.pdf]

Precision and bias of spatial capture–recapture estimates: A multi-site, multi-year Utah black bear case study

Greta M. Schmidt, Tabitha A. Graves, Jordan C. Pederson, Sarah L. Carroll

Ecological Applications

Appendix S1: Ecological Model Selection Results

Table S1. Full set of candidate models testing covariates hypothesized to influence the spatial scale parameter sigma at the five Utah black bear study areas. Highlighted models indicate the best-supported model for each study area that contained only informative parameters (i.e., 85% confidence interval of the coefficient estimate did not cover 0)

Site	model	logL	K	AIC	dAIC	CumWt
Kamas	D(~1) p(~1) sig(~1) asu(~1)	696.34	4	1,400.67	0.00	0.33
	D(~1) p(~1) sig(~session) asu(~1)	691.57	9	1,401.15	0.48	0.60
	D(~1) p(~1) sig(~sex + session + sex:session) asu(~1)	685.94	15	1,401.87	1.20	0.78
	D(~1) p(~1) sig(~sex) asu(~1)	696.33	5	1,402.67	1.99	0.90
	D(~1) p(~1) sig(~sex + session) asu(~1)	691.57	10	1,403.15	2.47	1.00

Site	model	logL	K	AIC	dAIC	CumWt
Boulder	D(~1) p(~1) sig(~session) asu(~1)	252.99	6	517.99	0.00	0.35
	D(~1) p(~1) sig(~1) asu(~1)	255.24	4	518.48	0.49	0.62
	D(~1) p(~1) sig(~sex + session) asu(~1)	252.58	7	519.17	1.18	0.81
	D(~1) p(~1) sig(~sex) asu(~1)	254.99	5	519.99	2.00	0.94
	D(~1) p(~1) sig(~sex + session + sex:session) asu(~1)	251.73	9	521.47	3.48	1.00
East Uinta	D(~1) p(~1) sig(~session) asu(~1)	165.29	6	342.58	0.00	0.60
	D(~1) p(~1) sig(~sex + session) asu(~1)	165.27	7	344.54	1.95	0.83
	D(~1) p(~1) sig(~1) asu(~1)	169.29	4	346.57	3.99	0.91

Site	model	logL	K	AIC	dAIC	CumWt
	D(~1) p(~1) sig(~sex + session + sex:session) asu(~1)	164.58	9	347.16	4.58	0.97
	D(~1) p(~1) sig(~sex) asu(~1)	169.28	5	348.57	5.99	1.00
Strawberry	D(~1) p(~1) sig(~session) asu(~1)	252.87	6	517.74	0.00	0.39
	D(~1) p(~1) sig(~1) asu(~1)	255.07	4	518.15	0.41	0.70
	D(~1) p(~1) sig(~sex + session) asu(~1)	252.86	7	519.72	1.99	0.85
	D(~1) p(~1) sig(~sex) asu(~1)	255.02	5	520.03	2.30	0.97
	D(~1) p(~1) sig(~sex + session + sex:session) asu(~1)	252.48	9	522.97	5.23	1.00
La Sal	D(~1) p(~1) sig(~session) asu(~1)	611.33	6	1,234.66	0.00	0.50

Site	model	logL	K	AIC	dAIC	CumWt
	D(~1) p(~1) sig(~sex + session + sex:session) asu(~1)	609.01	9	1,236.03	1.36	0.75
	D(~1) p(~1) sig(~sex + session) asu(~1)	611.07	7	1,236.15	1.48	0.99
	D(~1) p(~1) sig(~1) asu(~1)	617.30	4	1,242.60	7.94	0.99
	D(~1) p(~1) sig(~sex) asu(~1)	616.89	5	1,243.79	9.12	1.00

Table S2. Full set of candidate models testing covariates hypothesized to influence baseline detection probability at the five Utah black bear study areas. Highlighted models indicate the best-supported model for each study area that contained only informative parameters (i.e., 85% confidence interval of the coefficient estimate did not cover 0).

Site	model	logL	K	AIC	dAIC	CumWt
Kamas	D(~1) p(~avg_cov2 + b + scent) sig(~1) asu(~1)	601.33	6	1,214.66	0.00	0.66
	D(~1) p(~avg_cov2 + b) sig(~1) asu(~1)	603.13	5	1,216.26	1.59	0.95

Site	model	logL	K	AIC	dAIC	CumWt
	D(~1) p(~avg_cov2 + b + scent + session) sig(~1) asu(~1)	599.36	11	1,220.72	6.06	0.99
	D(~1) p(~avg_cov2 + b + session) sig(~1) asu(~1)	601.27	10	1,222.54	7.88	1.00
	D(~1) p(~avg_cov2 + scent) sig(~1) asu(~1)	609.28	5	1,228.56	13.89	1.00
	D(~1) p(~avg_cov2) sig(~1) asu(~1)	610.66	4	1,229.32	14.66	1.00
	D(~1) p(~avg_cov2 + session) sig(~1) asu(~1)	608.21	9	1,234.42	19.76	1.00
	D(~1) p(~b + scent) sig(~1) asu(~1)	616.90	5	1,243.79	29.13	1.00
	D(~1) p(~b) sig(~1) asu(~1)	618.97	4	1,245.93	31.27	1.00
	D(~1) p(~b + scent + session) sig(~1) asu(~1)	614.94	10	1,249.89	35.22	1.00

Site	model	logL	K	AIC	dAIC	CumWt
	D(~1) p(~b + session) sig(~1) asu(~1)	616.91	9	1,251.81	37.15	1.00
	D(~1) p(~scent) sig(~1) asu(~1)	628.37	4	1,264.75	50.09	1.00
	D(~1) p(~1) sig(~1) asu(~1)	629.96	3	1,265.92	51.25	1.00
	D(~1) p(~scent + session) sig(~1) asu(~1)	625.50	9	1,269.00	54.33	1.00
	D(~1) p(~session) sig(~1) asu(~1)	627.01	8	1,270.03	55.36	1.00
Boulder	D(~1) p(~session) sig(~1) asu(~1)	220.12	5	450.25	0.00	0.19
	D(~1) p(~scent + session) sig(~1) asu(~1)	219.35	6	450.70	0.46	0.35
	D(~1) p(~avg_cov2 + session) sig(~1) asu(~1)	219.55	6	451.09	0.84	0.47
	D(~1) p(~1) sig(~1) asu(~1)	223.02	3	452.04	1.79	0.55

Site	model	logL	K	AIC	dAIC	CumWt
	D(~1) p(~b + session) sig(~1) asu(~1)	220.12	6	452.25	2.00	0.62
	D(~1) p(~scent) sig(~1) asu(~1)	222.20	4	452.41	2.16	0.69
	D(~1) p(~b + scent + session) sig(~1) asu(~1)	219.34	7	452.67	2.42	0.74
	D(~1) p(~avg_cov2 + b + session) sig(~1) asu(~1)	219.55	7	453.09	2.84	0.79
	D(~1) p(~avg_cov2) sig(~1) asu(~1)	222.55	4	453.09	2.85	0.84
	D(~1) p(~avg_cov2 + scent) sig(~1) asu(~1)	221.77	5	453.54	3.29	0.87
	D(~1) p(~avg_cov2 + b + scent + session) sig(~1) asu(~1)	218.78	8	453.56	3.31	0.91
	D(~1) p(~b) sig(~1) asu(~1)	222.99	4	453.99	3.74	0.94

Site	model	logL	K	AIC	dAIC	CumWt
	D(~1) p(~b + scent) sig(~1) asu(~1)	222.12	5	454.23	3.99	0.97
	D(~1) p(~avg_cov2 + b) sig(~1) asu(~1)	222.50	5	455.01	4.76	0.98
	D(~1) p(~avg_cov2 + b + scent) sig(~1) asu(~1)	221.65	6	455.30	5.05	1.00
East Uinta	D(~1) p(~avg_cov2) sig(~session) asu(~1)	130.35	6	272.70	0.00	0.34
	D(~1) p(~avg_cov2 + session) sig(~session) asu(~1)	128.70	8	273.39	0.69	0.58
	D(~1) p(~avg_cov2 + b) sig(~session) asu(~1)	130.30	7	274.60	1.90	0.71
	D(~1) p(~avg_cov2 + scent) sig(~session) asu(~1)	130.34	7	274.68	1.98	0.84

Site	model	logL	K	AIC	dAIC	CumWt
	D(~1) p(~avg_cov2 + b + session) sig(~session) asu(~1)	128.80	9	275.61	2.91	0.92
	D(~1) p(~avg_cov2 + b + scent) sig(~session) asu(~1)	130.29	8	276.58	3.88	0.97
	D(~1) p(~avg_cov2 + b + scent + session) sig(~session) asu(~1)	128.71	10	277.42	4.72	1.00
	D(~1) p(~1) sig(~session) asu(~1)	138.37	5	286.75	14.05	1.00
	D(~1) p(~session) sig(~session) asu(~1)	136.80	7	287.60	14.89	1.00
	D(~1) p(~b + scent + session) sig(~session) asu(~1)	135.19	9	288.38	15.68	1.00
	D(~1) p(~b) sig(~session) asu(~1)	138.31	6	288.61	15.91	1.00

Site	model	logL	K	AIC	dAIC	CumWt
	D(~1) p(~scent) sig(~session) asu(~1)	138.32	6	288.65	15.95	1.00
	D(~1) p(~b + session) sig(~session) asu(~1)	136.35	8	288.69	15.99	1.00
	D(~1) p(~scent + session) sig(~session) asu(~1)	136.66	8	289.32	16.62	1.00
	D(~1) p(~b + scent) sig(~session) asu(~1)	138.24	7	290.48	17.77	1.00
Strawberry	D(~1) p(~avg_cov2 + b) sig(~1) asu(~1)	217.57	5	445.15	0.00	0.27
	D(~1) p(~avg_cov2) sig(~1) asu(~1)	219.08	4	446.17	1.02	0.43
	D(~1) p(~avg_cov2 + b + scent) sig(~1) asu(~1)	217.10	6	446.19	1.05	0.59
	D(~1) p(~avg_cov2 + b + session) sig(~1) asu(~1)	216.11	7	446.21	1.06	0.75

Site	model	logL	K	AIC	dAIC	CumWt
	D(~1) p(~avg_cov2 + b + scent + session) sig(~1) asu(~1)	215.64	8	447.29	2.14	0.84
	D(~1) p(~avg_cov2 + scent) sig(~1) asu(~1)	218.66	5	447.33	2.18	0.93
	D(~1) p(~avg_cov2 + session) sig(~1) asu(~1)	217.90	6	447.80	2.65	1.00
	D(~1) p(~b) sig(~1) asu(~1)	229.76	4	467.52	22.37	1.00
	D(~1) p(~b + scent) sig(~1) asu(~1)	229.19	5	468.38	23.24	1.00
	D(~1) p(~b + session) sig(~1) asu(~1)	228.64	6	469.27	24.13	1.00
	D(~1) p(~b + scent + session) sig(~1) asu(~1)	228.09	7	470.18	25.04	1.00
	D(~1) p(~1) sig(~1) asu(~1)	232.87	3	471.74	26.60	1.00

Site	model	logL	K	AIC	dAIC	CumWt
	D(~1) p(~scent) sig(~1) asu(~1)	232.36	4	472.71	27.57	1.00
	D(~1) p(~session) sig(~1) asu(~1)	232.09	5	474.18	29.03	1.00
	D(~1) p(~scent + session) sig(~1) asu(~1)	231.57	6	475.15	30.00	1.00
La Sal	D(~1) p(~avg_cov2 + b + scent) sig(~session) asu(~1)	463.84	8	943.68	0.00	0.53
	D(~1) p(~b + scent) sig(~session) asu(~1)	466.09	7	946.18	2.50	0.69
	D(~1) p(~avg_cov2 + b + scent + session) sig(~session) asu(~1)	463.40	10	946.80	3.12	0.80
	D(~1) p(~avg_cov2 + b) sig(~session) asu(~1)	466.40	7	946.80	3.12	0.91

Site	model	logL	K	AIC	dAIC	CumWt
	D(~1) p(~b + scent + session) sig(~session) asu(~1)	465.64	9	949.27	5.60	0.94
	D(~1) p(~b) sig(~session) asu(~1)	468.74	6	949.48	5.80	0.97
	D(~1) p(~avg_cov2 + b + session) sig(~session) asu(~1)	466.05	9	950.10	6.42	0.99
	D(~1) p(~b + session) sig(~session) asu(~1)	468.37	8	952.75	9.07	1.00
	D(~1) p(~avg_cov2 + scent) sig(~session) asu(~1)	472.00	7	957.99	14.32	1.00
	D(~1) p(~avg_cov2) sig(~session) asu(~1)	474.09	6	960.18	16.51	1.00
	D(~1) p(~scent) sig(~session) asu(~1)	474.68	6	961.35	17.68	1.00

Site	model	logL	K	AIC	dAIC	CumWt
	D(~1) p(~avg_cov2 + session) sig(~session) asu(~1)	473.62	8	963.25	19.57	1.00
	D(~1) p(~1) sig(~session) asu(~1)	476.86	5	963.72	20.05	1.00
	D(~1) p(~scent + session) sig(~session) asu(~1)	474.18	8	964.36	20.68	1.00
	D(~1) p(~session) sig(~session) asu(~1)	476.30	7	966.61	22.93	1.00

Table S3. Full set of candidate models testing covariates hypothesized to influence density at the five Utah black bear study areas. Highlighted models indicate the best-supported model for each study area that contained only informative parameters (i.e., 85% confidence interval of the coefficient estimate did not cover 0).

Site	model	logL	K	AIC	dAIC	CumWt
Kamas	D(~elev.scaled) p(~avg_cov2 + b) sig(~1) asu(~1)	601.43	6	1,214.85	0.00	0.43
	D(~1) p(~avg_cov2 + b) sig(~1) asu(~1)	603.13	5	1,216.26	1.41	0.65

Site	model	logL	K	AIC	dAIC	CumWt
	D(~cov.scaled + elev.scaled) p(~avg_cov2 + b) sig(~1) asu(~1)	601.42	7	1,216.85	2.00	0.81
	D(~cov.scaled) p(~avg_cov2 + b) sig(~1) asu(~1)	602.85	6	1,217.69	2.84	0.91
	D(~elev.scaled + session) p(~avg_cov2 + b) sig(~1) asu(~1)	598.79	11	1,219.58	4.73	0.95
	D(~session) p(~avg_cov2 + b) sig(~1) asu(~1)	600.41	10	1,220.82	5.97	0.98
	D(~cov.scaled + elev.scaled + session) p(~avg_cov2 + b) sig(~1) asu(~1)	598.79	12	1,221.57	6.72	0.99
	D(~cov.scaled + session) p(~avg_cov2 + b) sig(~1) asu(~1)	600.33	11	1,222.66	7.81	1.00

Site	model	logL	K	AIC	dAIC	CumWt
Boulder	D(~elev.scaled) p(~1) sig(~1) asu(~1)	219.54	4	447.07	0.00	0.48
	D(~cov.scaled + elev.scaled) p(~1) sig(~1) asu(~1)	219.38	5	448.76	1.69	0.69
	D(~elev.scaled + session) p(~1) sig(~1) asu(~1)	218.67	6	449.35	2.27	0.85
	D(~cov.scaled + elev.scaled + session) p(~1) sig(~1) asu(~1)	218.54	7	451.09	4.01	0.91
	D(~1) p(~1) sig(~1) asu(~1)	223.02	3	452.04	4.97	0.95
	D(~session) p(~1) sig(~1) asu(~1)	221.64	5	453.27	6.20	0.98
	D(~cov.scaled) p(~1) sig(~1) asu(~1)	222.98	4	453.96	6.89	0.99
	D(~cov.scaled + session) p(~1) sig(~1) asu(~1)	221.57	6	455.14	8.07	1.00

**Site**	**model**	**logL**	**K**	**AIC**	**dAIC**	**CumWt**
East Uinta	D(~cov.scaled) p(~avg_cov2) sig(~session) asu(~1)	125.50	7	265.00	0.00	0.58
	D(~cov.scaled + elev.scaled) p(~avg_cov2) sig(~session) asu(~1)	125.46	8	266.92	1.92	0.81
	D(~cov.scaled + session) p(~avg_cov2) sig(~session) asu(~1)	125.08	9	268.15	3.15	0.93
	D(~cov.scaled + elev.scaled + session) p(~avg_cov2) sig(~session) asu(~1)	125.04	10	270.07	5.07	0.98
	D(~1) p(~avg_cov2) sig(~session) asu(~1)	130.35	6	272.70	7.70	0.99
	D(~elev.scaled) p(~avg_cov2) sig(~session) asu(~1)	129.80	7	273.61	8.60	1.00

Site	model	logL	K	AIC	dAIC	CumWt
	D(~session) p(~avg_cov2) sig(~session) asu(~1)	129.92	8	275.83	10.83	1.00
	D(~elev.scaled + session) p(~avg_cov2) sig(~session) asu(~1)	129.43	9	276.86	11.86	1.00
Strawberry	D(~session) p(~avg_cov2) sig(~1) asu(~1)	216.57	6	445.14	0.00	0.21
	D(~cov.scaled + session) p(~avg_cov2) sig(~1) asu(~1)	215.62	7	445.24	0.10	0.41
	D(~1) p(~avg_cov2) sig(~1) asu(~1)	219.08	4	446.17	1.03	0.54
	D(~cov.scaled) p(~avg_cov2) sig(~1) asu(~1)	218.14	5	446.29	1.15	0.66
	D(~elev.scaled + session) p(~avg_cov2) sig(~1) asu(~1)	216.21	7	446.42	1.29	0.77

Site	model	logL	K	AIC	dAIC	CumWt
	D(~cov.scaled + elev.scaled + session) p(~avg_cov2) sig(~1) asu(~1)	215.29	8	446.57	1.44	0.87
	D(~elev.scaled) p(~avg_cov2) sig(~1) asu(~1)	218.74	5	447.48	2.34	0.94
	D(~cov.scaled + elev.scaled) p(~avg_cov2) sig(~1) asu(~1)	217.83	6	447.65	2.51	1.00
La Sal	D(~elev.scaled) p(~avg_cov2 + b + scent) sig(~session) asu(~1)	461.38	9	940.76	0.00	0.37
	D(~elev.scaled + session) p(~avg_cov2 + b + scent) sig(~session) asu(~1)	459.90	11	941.80	1.04	0.59

Site	model	logL	K	AIC	dAIC	CumWt
	D(~cov.scaled + elev.scaled) p(~avg_cov2 + b + scent) sig(~session) asu(~1)	461.37	10	942.74	1.98	0.73
	D(~1) p(~avg_cov2 + b + scent) sig(~session) asu(~1)	463.84	8	943.68	2.91	0.81
	D(~cov.scaled + elev.scaled + session) p(~avg_cov2 + b + scent) sig(~session) asu(~1)	459.89	12	943.78	3.02	0.90
	D(~session) p(~avg_cov2 + b + scent) sig(~session) asu(~1)	462.46	10	944.92	4.16	0.94
	D(~cov.scaled) p(~avg_cov2 + b + scent) sig(~session) asu(~1)	463.68	9	945.35	4.59	0.98

Site	model	logL	K	AIC	dAIC	CumWt
	D(~cov.scaled + session) p(~avg_cov2 + b + scent) sig(~session) asu(~1)	462.31	11	946.63	5.86	1.00

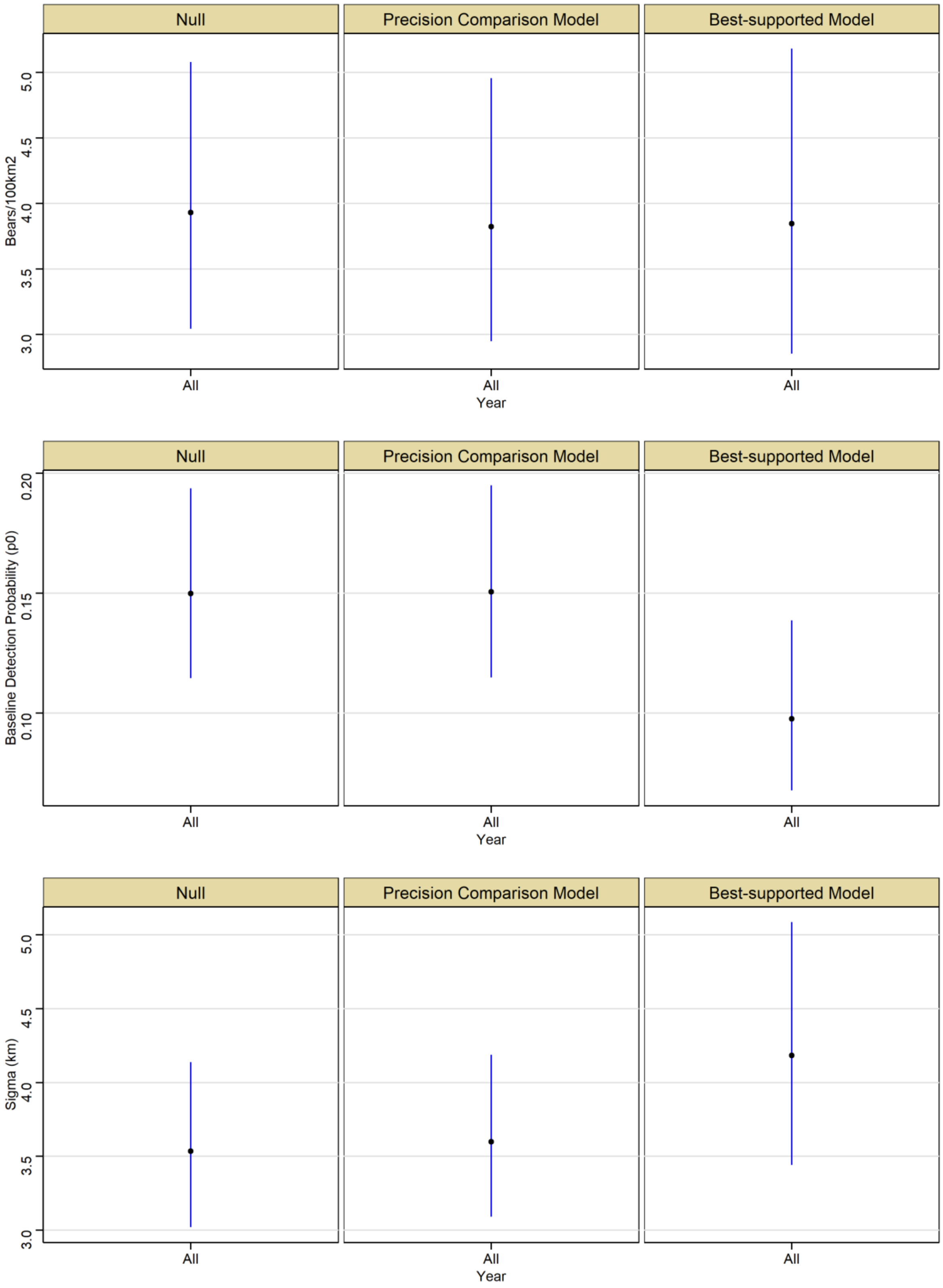

Figure S1. Density, detection, and sigma estimates for the null, precision comparison model, and Best-supported Model model for the Kamas study area

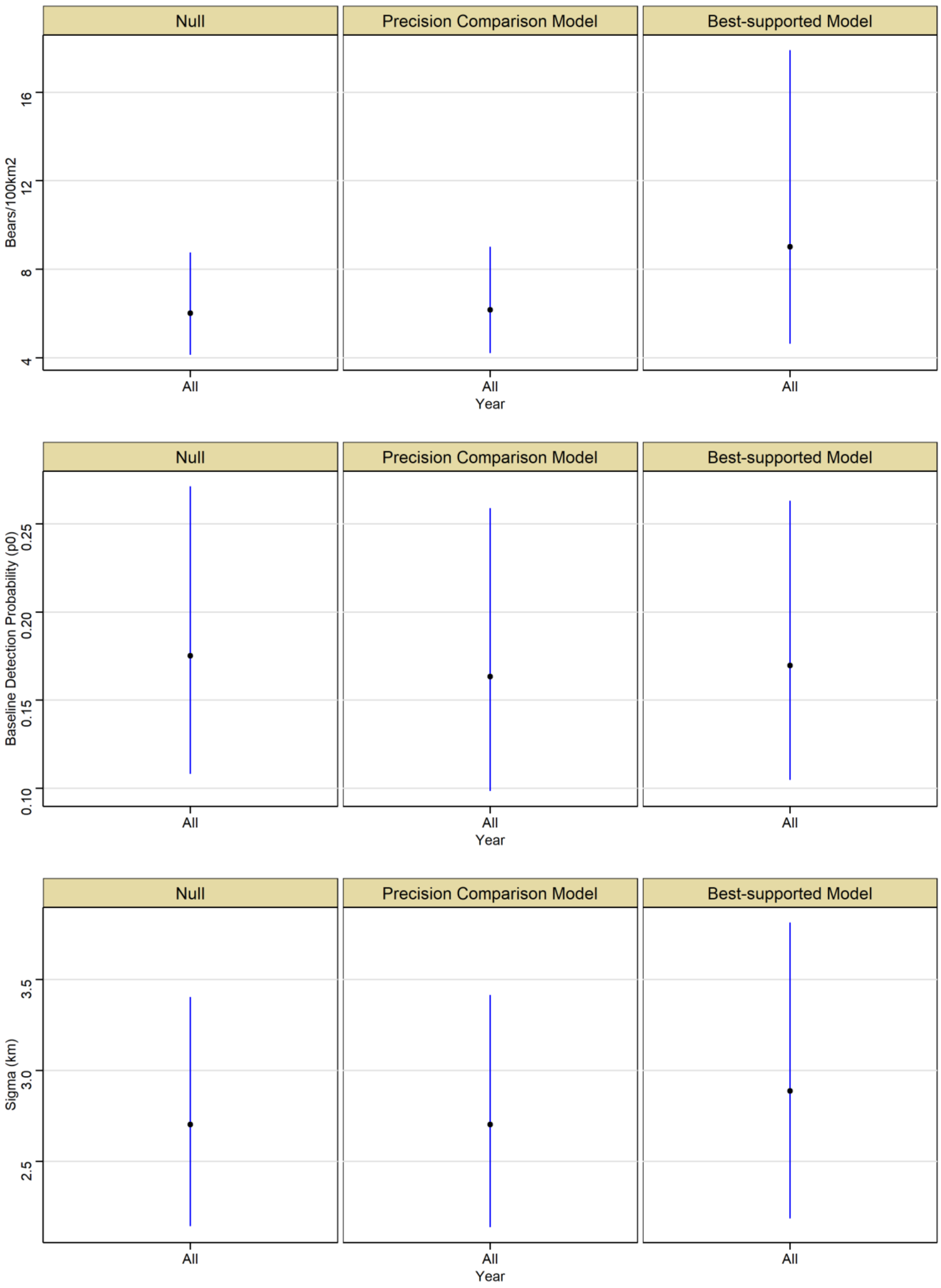

Figure S2. Density, detection, and sigma estimates for the null, precision comparison model, and best-supported model model for the Boulder study area

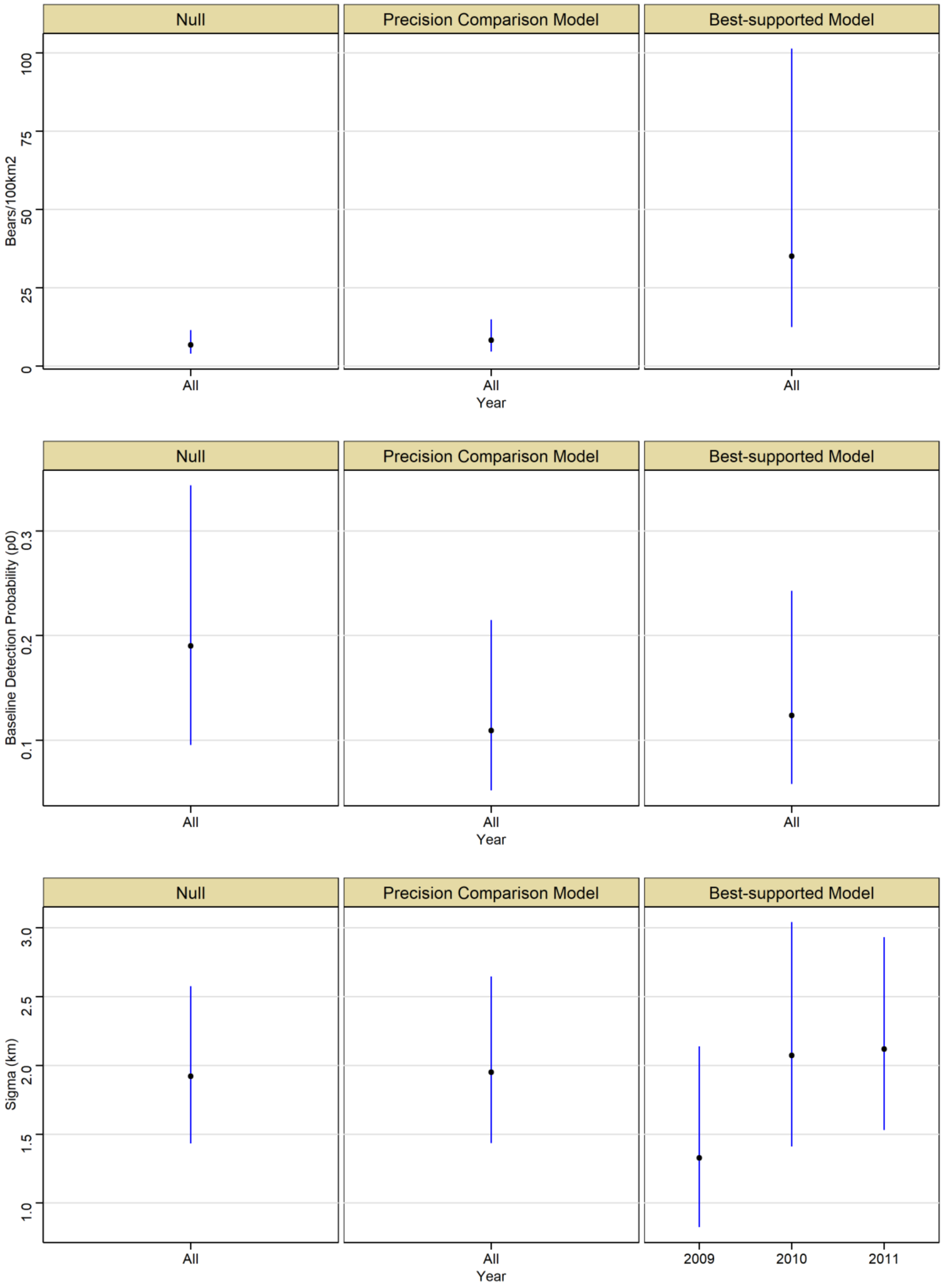

Figure S3. Density, detection, and sigma estimates for the null, precision comparison model, and Best-supported Model model for the East Uinta study area

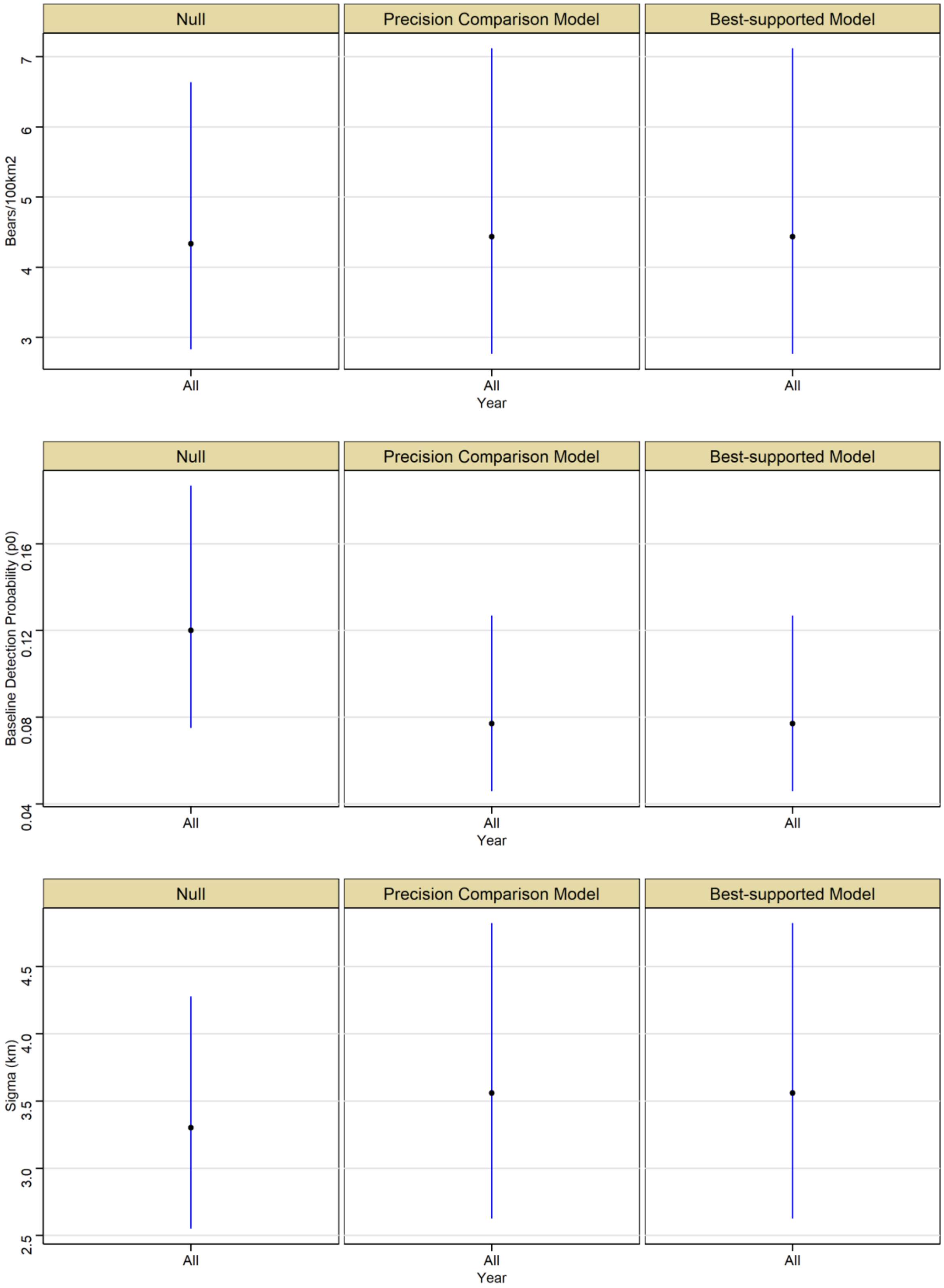

Figure S4. Density, detection, and sigma estimates for the null, precision comparison model, and Best-supported Model model for the Strawberry study area

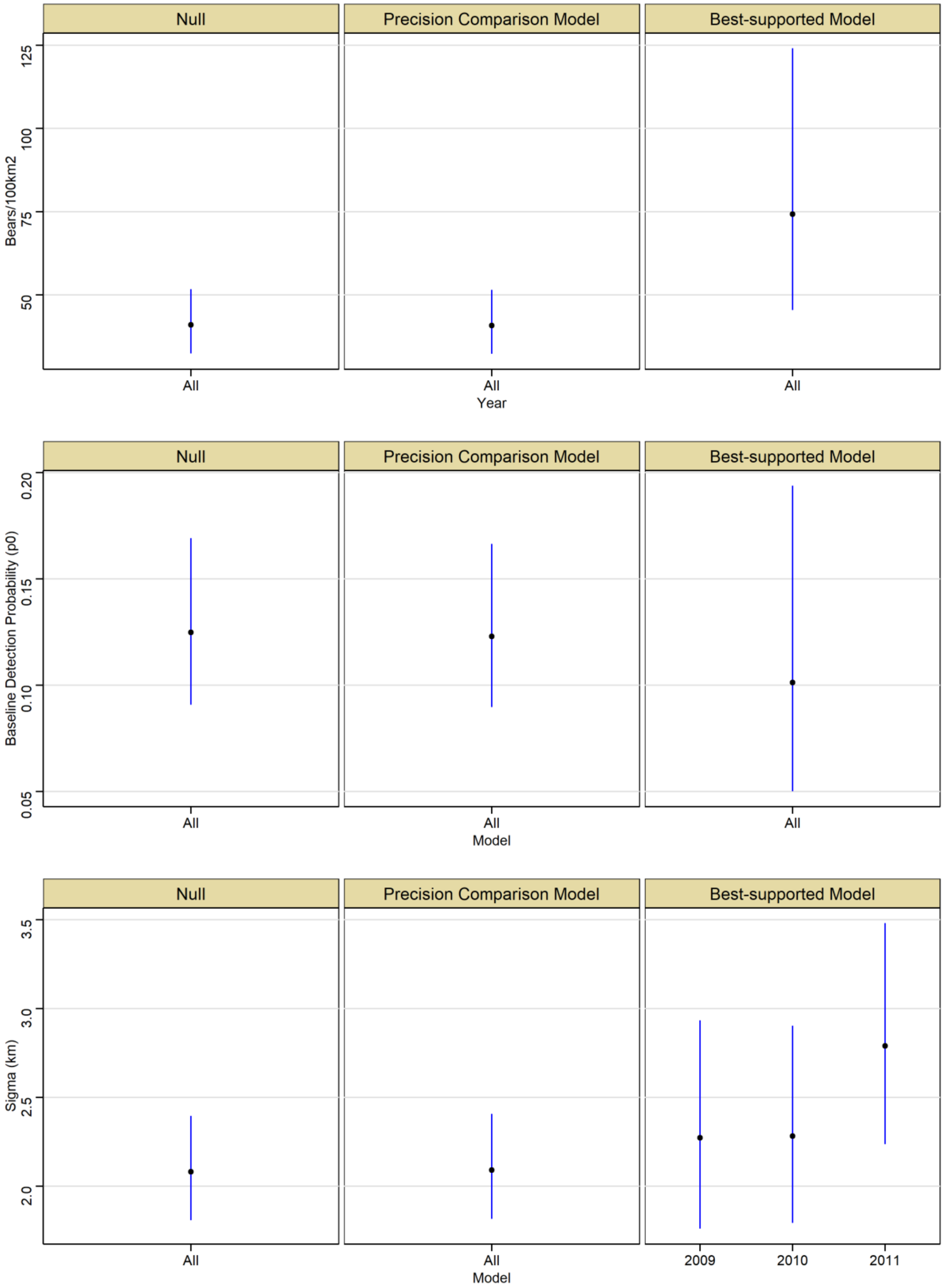

Figure S5. Density, detection, and sigma estimates for the null, precision comparison model, and Best-supported Model model for the La Sal study area
