# Supplementary material for: Precision and bias of spatial capture–recapture estimates: A multi‐site, multi‐year Utah black bear case study
Source: Ecol Appl. 2022 May 17;32(5):e2618. doi: 10.1002/eap.2618 (PMC9287071; doi:10.1002/eap.2618)
Supplement: Supplementary file 2 — Appendix S2 [file EAP-32-0-s001.pdf]

# Precision and bias of spatial capture–recapture estimates: A multi-site, multi-year Utah black bear case study


## Greta M. Schmidt, Tabitha A. Graves, Jordan C. Pederson, Sarah L. Carroll



## Ecological Applications


## Appendix S2: SCR Simulations - Exploring Potential Sources of Bias

*Section S1: Summarizing and Evaluating Simulations*

Summary statistics from the 500 simulated datasets for the six simulation scenarios for density, detection (p0 and pb), and sigma are detailed in Table S1. Distributions of the summary statistics for these six scenarios are visualized in Figure S1, and include lines for comparison with the summarized attributes of the sampling data collected from the La Sal site during each of the three years of sampling, as well as an additional dotted line representing all three years of data combined. La Sal sampling datasets appear to be a reasonable outcome of our simulated density of 60 bears/km$^2$, with a trap-happy effect on detection and sigma of 2km. There were issues with consistent model convergence at simulated densities of 2 bears/100km$^2$, particularly when a behavioral covariate on detection was used to simulate capture–recapture datasets.

To quantify bias and precision of the resulting parameter estimates for density, detection probability, and sigma, we evaluated average deviation of estimates from the true value used to simulate datasets, as well as whether the true value had coverage by the 95% confidence intervals (CIC). Density estimates for all scenarios largely had confidence interval coverage of the true value (Table S2), and density estimates were generally unbiased (Table S3). Negative bias in density was most common when behavior was used to simulate the data and then not modeled (Table S3). Detection estimates were also largely unbiased (Table S2, Table S4). Sigma estimates were sometimes positively biased (Table S2, Table S5). For example, at 60 bears/100 km$^2$, all 500 sigma estimates from datasets simulated without a behavioral covariate were positively biased. However, positive bias in sigma did not appear to influence bias in either the density or detection estimates.

We looked at the relationship between estimates for density, detection, and sigma versus the ratio of individuals with spatial recaptures relative to all recaptures (i.e., spatial recaptured + recaptured only at trap of first detection, Figure S2). Because of convergence issues at lower densities, we focused on 60 bears/100 km$^2$. Though estimate confidence intervals cover the true value, estimates appear biased high when the ratio of spatially recaptured individuals to all recaptured individuals falls below 0.3. Density estimates with a ratio of 0.3 or less were biased high by an average of 49.97 bears/100km$^2$ (average of 109.97 ± 43.68 bears/100 km$^2$). Density estimates above this ratio were biased from the true value an average of -2.29 bears/100km$^2$ (average of 57.71 ± 18.60 bears/100 km$^2$). This suggests that datasets with a low ratio of

spatially recaptured individuals to total recaptured individuals have the potential to introduce positive bias in density estimates.

To evaluate relationships of precision and bias among all three parameter estimates, we visualized each collection of parameter estimates from our simulations three times, denoting which estimates were associated with confidence interval coverage in density, detection, and sigma (Figure S3). There did not appear to be coincident bias, for example biased density estimates were not necessarily associated with biased detection and sigma estimates.

We observed the same relationships with precision of SCR parameter estimates, defined as the coefficient of variation (CV, $SE_{Estimate}$/Estimate), that we observed in our empirical datasets (Figure S4).

***Table 1****: Table S1. Mean (SD) sampling data summary statistics from the 500 simulated datasets for the nine models fit for the six simulation scenarios.*

| True Density (Bears/100km2) | pb simulated? | pb modeled? | # Indiv | # Dets | # Recaps | # Spatial Recaps | Spatial Recap Indiv/ All Recap Indiv |
|---|---|---|---|---|---|---|---|
| 2 | Yes | No | 4.15 (1.56) | 6.45 (2.32) | 2.3 (1.25) | 1.6 (0.92) | 0.86 (0.22) |
| 2 | Yes | Yes | 4.15 (1.58) | 6.52 (2.34) | 2.37 (1.25) | 1.62 (0.93) | 0.85 (0.23) |
| 2 | No | No | 5.8 (2.09) | 9.72 (4.1) | 3.92 (2.56) | 3.04 (1.93) | 0.91 (0.17) |
| 20 | Yes | No | 34.39 (5.7) | 47.35 (8.61) | 12.96 (4.34) | 8.1 (3.16) | 0.71 (0.15) |
| 20 | Yes | Yes | 34.41 (5.71) | 47.44 (8.59) | 13.03 (4.29) | 8.13 (3.15) | 0.7 (0.15) |
| 20 | No | No | 56.66 (7.1) | 94.12 (13.54) | 37.46 (8.32) | 27.57 (6.52) | 0.84 (0.08) |
| 60 | Yes | No | 62.7 (7.7) | 78.29 (10.24) | 15.58 (4.33) | 7.69 (2.8) | 0.55 (0.14) |
| 60 | Yes | Yes | 62.7 (7.7) | 78.29 (10.24) | 15.58 (4.33) | 7.69 (2.8) | 0.55 (0.14) |
| 60 | No | No | 170.3 (12.31) | 283.41 (24.22) | 113.11 (14.62) | 83.56 (11.22) | 0.85 (0.04) |

***Table 2***: *Table S2. Summary of whether estimates from the nine models fit for the six simulation scenarios for density, baseline detection, detection with behavior, and sigma had confidence interval coverage of the true value (Yes, No: Above True Value, No: Below True Value)*

| True Density (Bears/100 km2) | pb simulated? | pb modeled? | D | p0 | pbehav | sigma |
|---|---|---|---|---|---|---|
| 2 | Yes | No | (229,0,6) | (205,29,1) | - | (235,0,0) |
| 2 | Yes | Yes | (210,1,8) | (195,13,4) | (164,2,0) | (199,7,13) |
| 2 | No | No | (419,4,11) | (434,0,0) | - | (399,34,1) |
| 20 | Yes | No | (461,5,33) | (366,132,1) | - | (393,102,4) |
| 20 | Yes | Yes | (475,1,19) | (468,18,9) | (480,13,1) | (381,105,9) |
| 20 | No | No | (481,8,11) | (500,0,0) | - | (59,441,0) |
| 60 | Yes | No | (418,0,82) | (288,211,1) | - | (387,6,107) |
| 60 | Yes | Yes | (469,0,31) | (467,25,8) | (483,10,7) | (456,9,35) |
| 60 | No | No | (479,8,13) | (500,0,0) | - | (0,500,0) |

***Table 3**: Table S3. Mean and standard deviation of density estimates, and mean difference of estimated density - true density for the nine models fit for the six simulation scenarios*

| True Density (Bears/100km2) | pb simulated? | pb modeled? | Mean (SD) Density Estimate (Bears/100km2) | Mean Estimated Density - True Density (Bears/100km2) |
|---|---|---|---|---|
| 2 | Yes | No | 1.86 (1.26) | -0.14 |
| 2 | Yes | Yes | 1.69 (1.12) | -0.31 |
| 2 | No | No | 2.3 (1.44) | 0.30 |
| 20 | Yes | No | 18.95 (6.34) | -1.05 |
| 20 | Yes | Yes | 20.47 (7.39) | 0.47 |
| 20 | No | No | 19.95 (3.57) | -0.05 |
| 60 | Yes | No | 50.31 (12.47) | -9.69 |
| 60 | Yes | Yes | 60.01 (22.94) | 0.01 |
| 60 | No | No | 58.99 (5.67) | -1.01 |

***Table 4****: Table S4. Mean and standard deviation of detection estimates, and mean difference of estimated detection - true detection for the nine models fit for the six simulation scenarios*

| **True Density (Bears/100km2)** | **pb simulated?** | **pb modeled?** | **True p0** | **Mean (SD) p0 Estimate** | **Mean p0 Estimate - True p0** | **True pb** | **Mean (SD) pb Estimate** | **Mean Estimated pb - True pb** |
|---|---|---|---|---|---|---|---|---|
| 2 | Yes | No | 0.07 | 0.18 (0.14) | 0.11 | 0.15 | - | - |
| 2 | Yes | Yes | 0.07 | 0.3 (0.29) | 0.23 | 0.15 | 0.18 (0.21) | 0.03 |
| 2 | No | No | 0.15 | 0.17 (0.1) | 0.02 | - | - | - |
| 20 | Yes | No | 0.07 | 0.12 (0.05) | 0.05 | 0.15 | - | - |
| 20 | Yes | Yes | 0.07 | 0.1 (0.09) | 0.03 | 0.15 | 0.15 (0.07) | 0 |
| 20 | No | No | 0.15 | 0.15 (0.03) | 0.00 | - | - | - |
| 60 | Yes | No | 0.07 | 0.13 (0.05) | 0.06 | 0.15 | - | - |
| 60 | Yes | Yes | 0.07 | 0.1 (0.08) | 0.03 | 0.15 | 0.15 (0.05) | 0 |
| 60 | No | No | 0.15 | 0.15 (0.02) | 0.00 | - | - | - |

***Table 5**: Table S5. Mean and standard deviation of sigma estimates, and mean difference of estimated sigma - true sigma for the nine models fit for the six simulation scenarios*

| True Density (Bears/100km2) | pb simulated? | pb modeled? | True Sigma (km) | Mean (SD) Sigma Estimate (km) | Mean Sigma Estimate (km) - True Sigma (km) |
|---|---|---|---|---|---|
| 2 | Yes | No | 2 | 36.17 (296.45) | 34.17 |
| 2 | Yes | Yes | 2 | 15.71 (175.33) | 13.71 |
| 2 | No | No | 2 | 16.99 (158.84) | 14.99 |
| 20 | Yes | No | 2 | 2.53 (0.59) | 0.53 |
| 20 | Yes | Yes | 2 | 2.82 (0.89) | 0.82 |
| 20 | No | No | 2 | 2.82 (0.31) | 0.82 |
| 60 | Yes | No | 2 | 1.86 (0.34) | -0.14 |
| 60 | Yes | Yes | 2 | 2.11 (0.51) | 0.11 |
| 60 | No | No | 2 | 2.83 (0.17) | 0.83 |

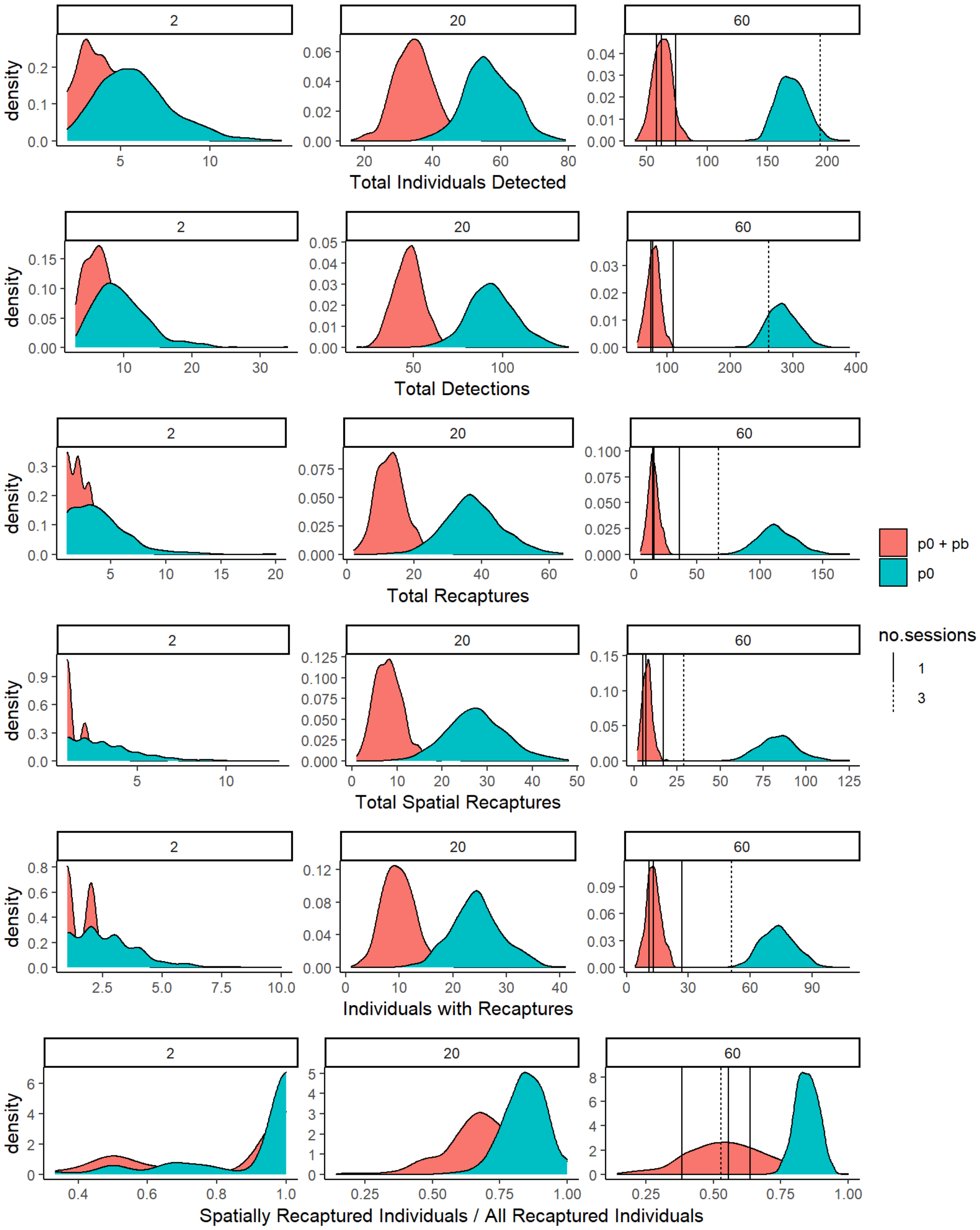


*Figure S1. Distribution of selected sampling data attributes for the 500 simulations for each of the 6 simulation scenarios: 3 densities (2,20,60 bears/100km²), each simulated with (p0 + pb) and without (p0) a trap-happy effect on behavior. Black lines represent the sampling data attributes from each of the three years of data collected at the La Sal site*

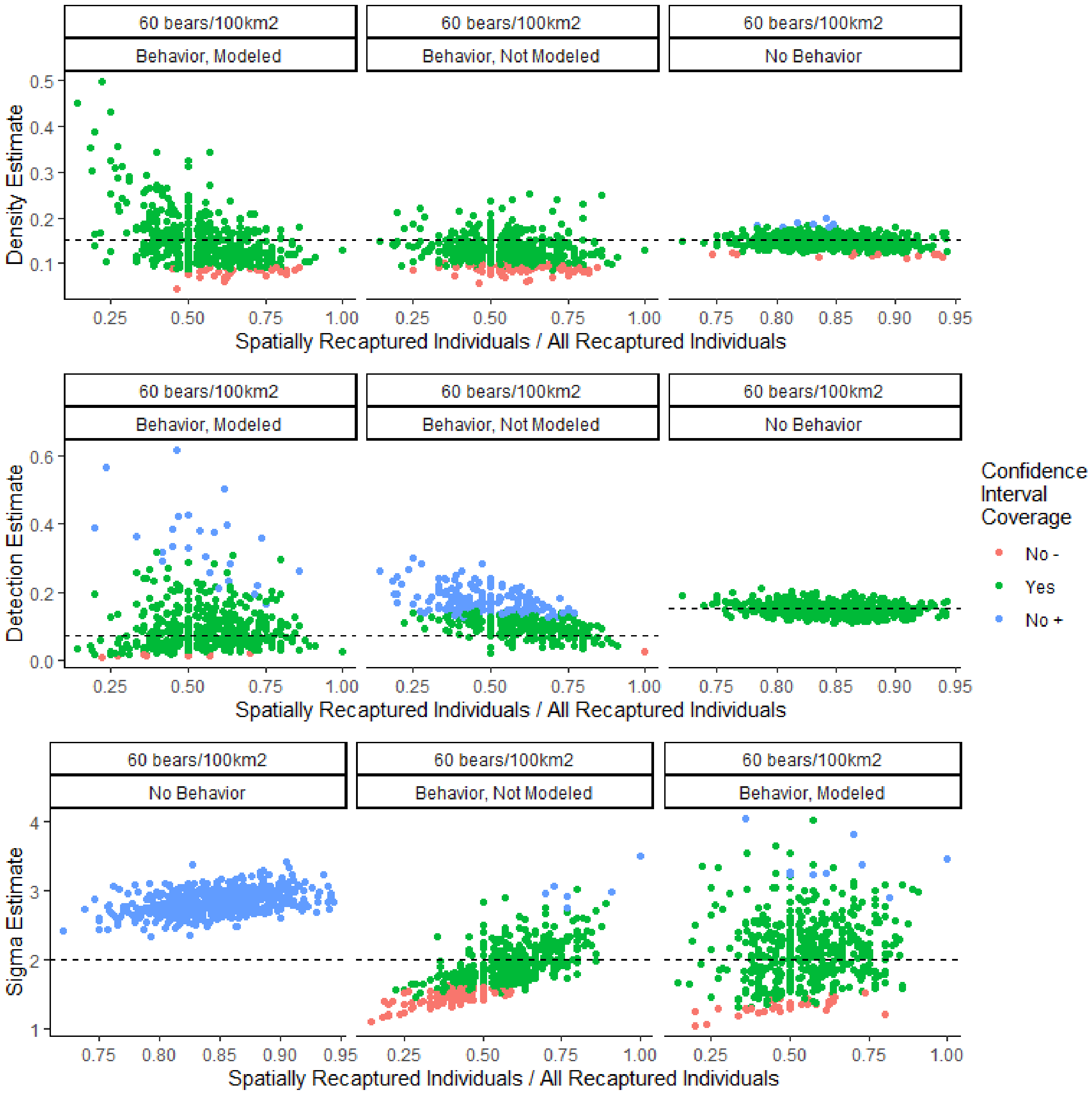


*Figure S2. Ratio of individuals with spatial recaptures to all recaptured individuals compared to estimates for density, detection and sigma, with the true parameter value indicated by the dashed black line. Color of the estimate indicates confidence interval coverage for that parameter was below the true value (red), encompassed the true value (green), or was above the true value (blue)*

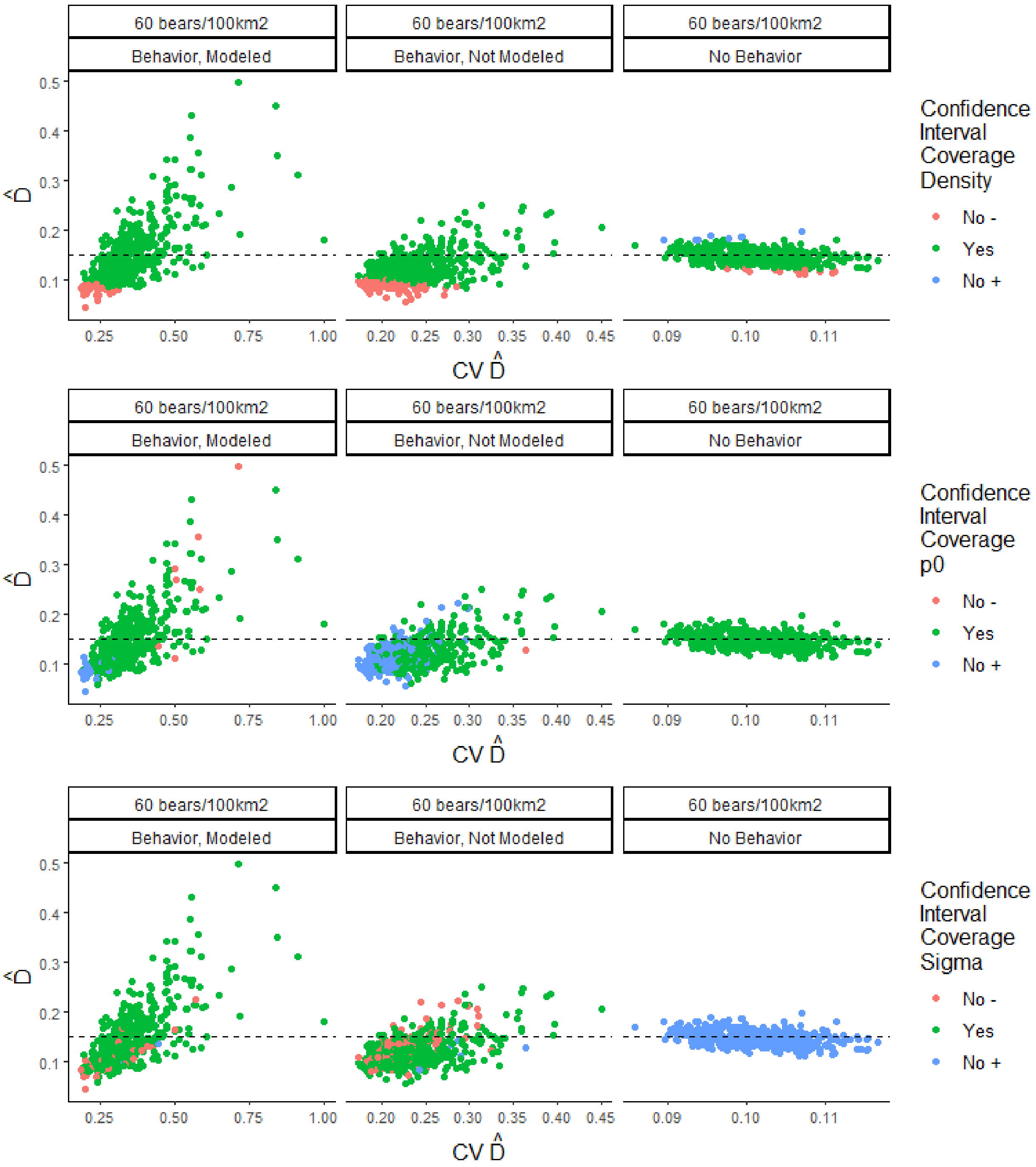
60 bears/100km2
Behavior, Modeled
Behavior, Not Modeled
No Behavior
D̂
CV D̂
0.1
0.2
0.3
0.4
0.5
0.25
0.50
0.75
1.00
0.20
0.25
0.30
0.35
0.40
0.45
0.09
0.10
0.11
Confidence Interval Coverage Density
Confidence Interval Coverage p0
Confidence Interval Coverage Sigma
No -
Yes
No +

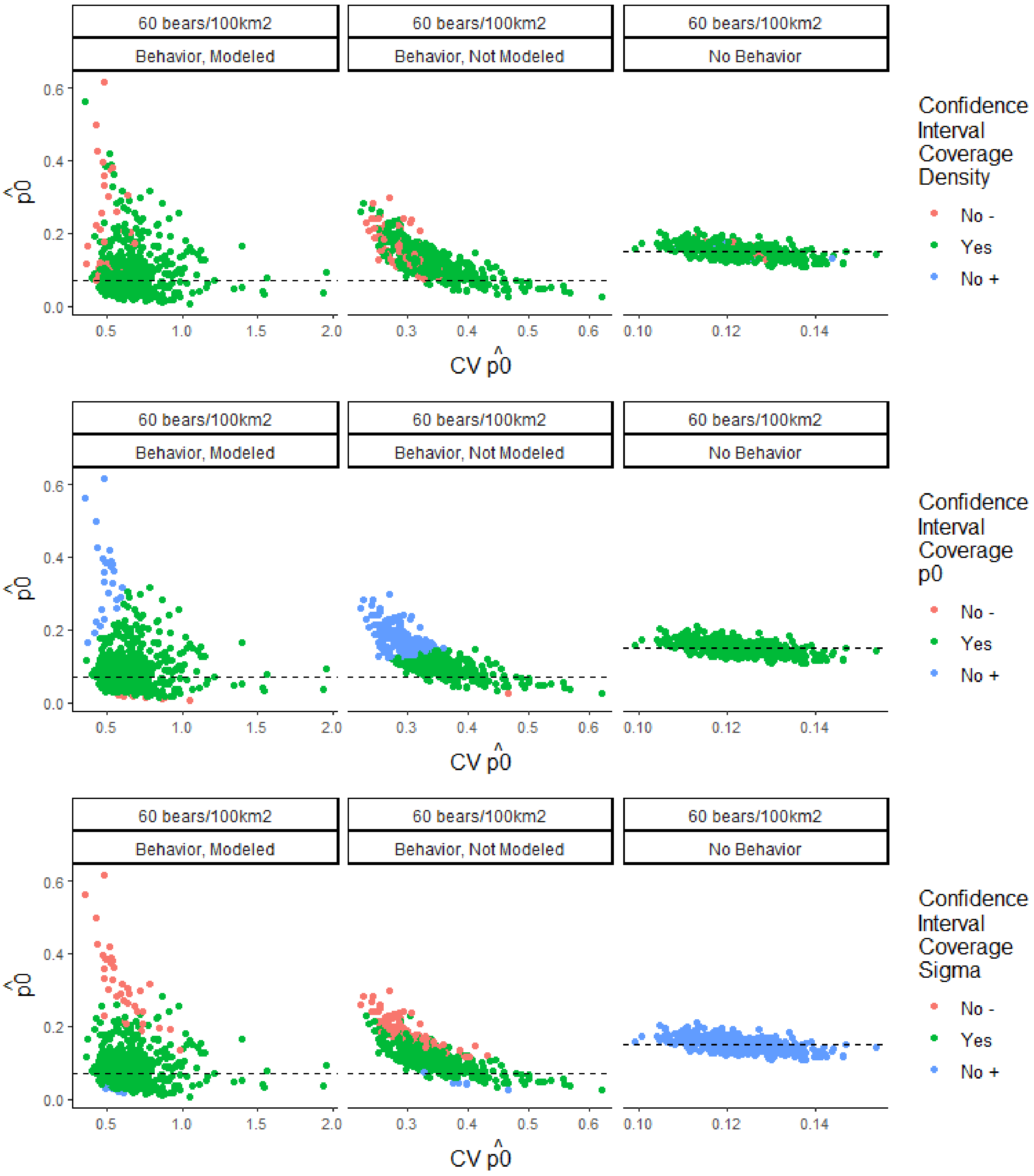
60 bears/100km2
Behavior, Modeled
60 bears/100km2
Behavior, Not Modeled
60 bears/100km2
No Behavior
0.6
0.4
0.2
0.0
^p0
0.5
1.0
1.5
2.0
0.3
0.4
0.5
0.6
0.10
0.12
0.14
CV ^p0
Confidence Interval Coverage Density
No -
Yes
No +
60 bears/100km2
Behavior, Modeled
60 bears/100km2
Behavior, Not Modeled
60 bears/100km2
No Behavior
Confidence Interval Coverage p0
No -
Yes
No +
60 bears/100km2
Behavior, Modeled
60 bears/100km2
Behavior, Not Modeled
60 bears/100km2
No Behavior
Confidence Interval Coverage Sigma
No -
Yes
No +

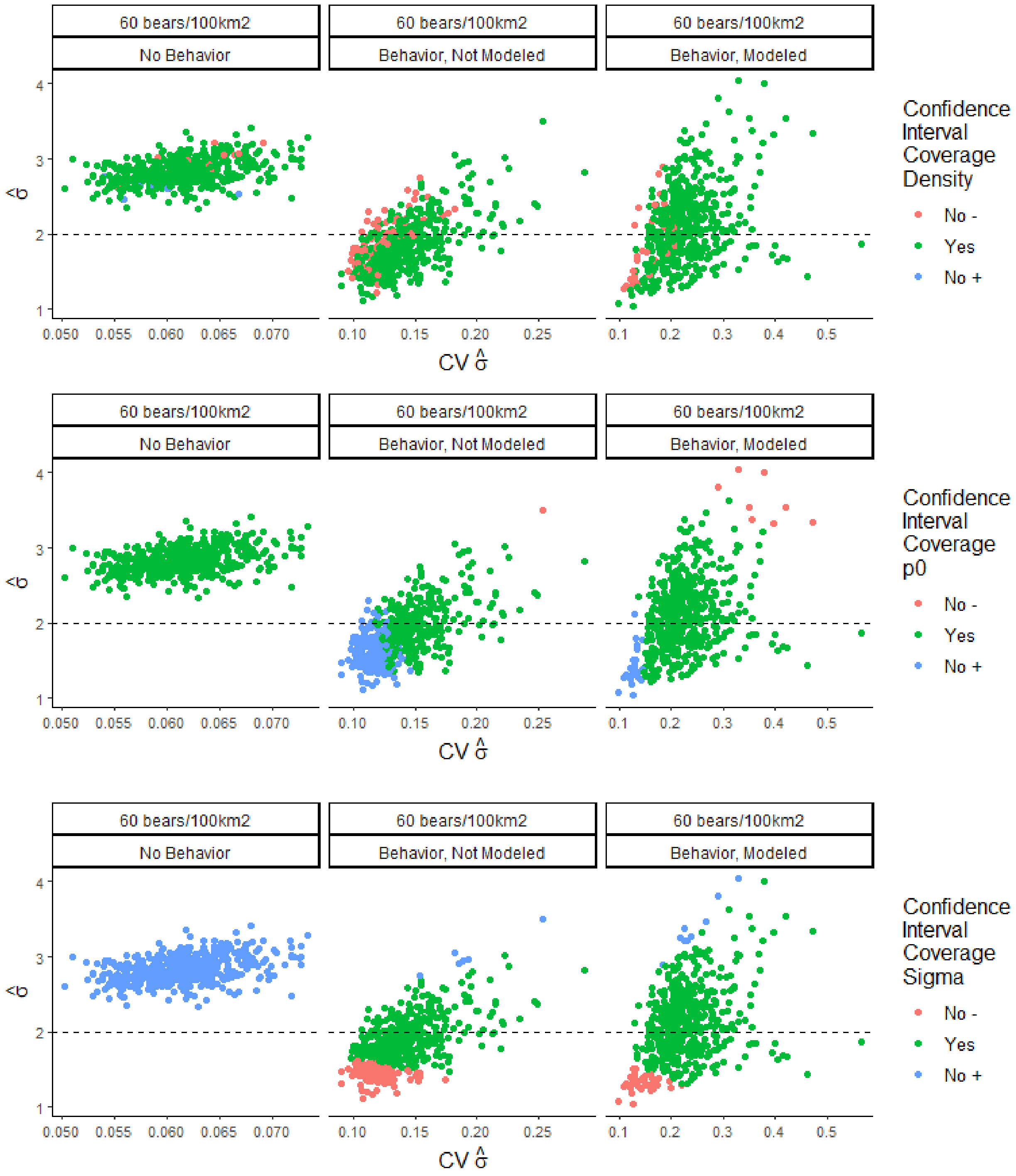


*Figure S3. The coefficient of variation (CV, $SE_{Estimate}$/Estimate) for estimates of density, detection, and sigma versus the parameter estimate, with the true parameter value indicated by the dashed black line. For each parameter we visualized confidence interval coverage across all three parameter estimates (Red = Negatively biased, Green = No bias, Blue = Positively biased)*

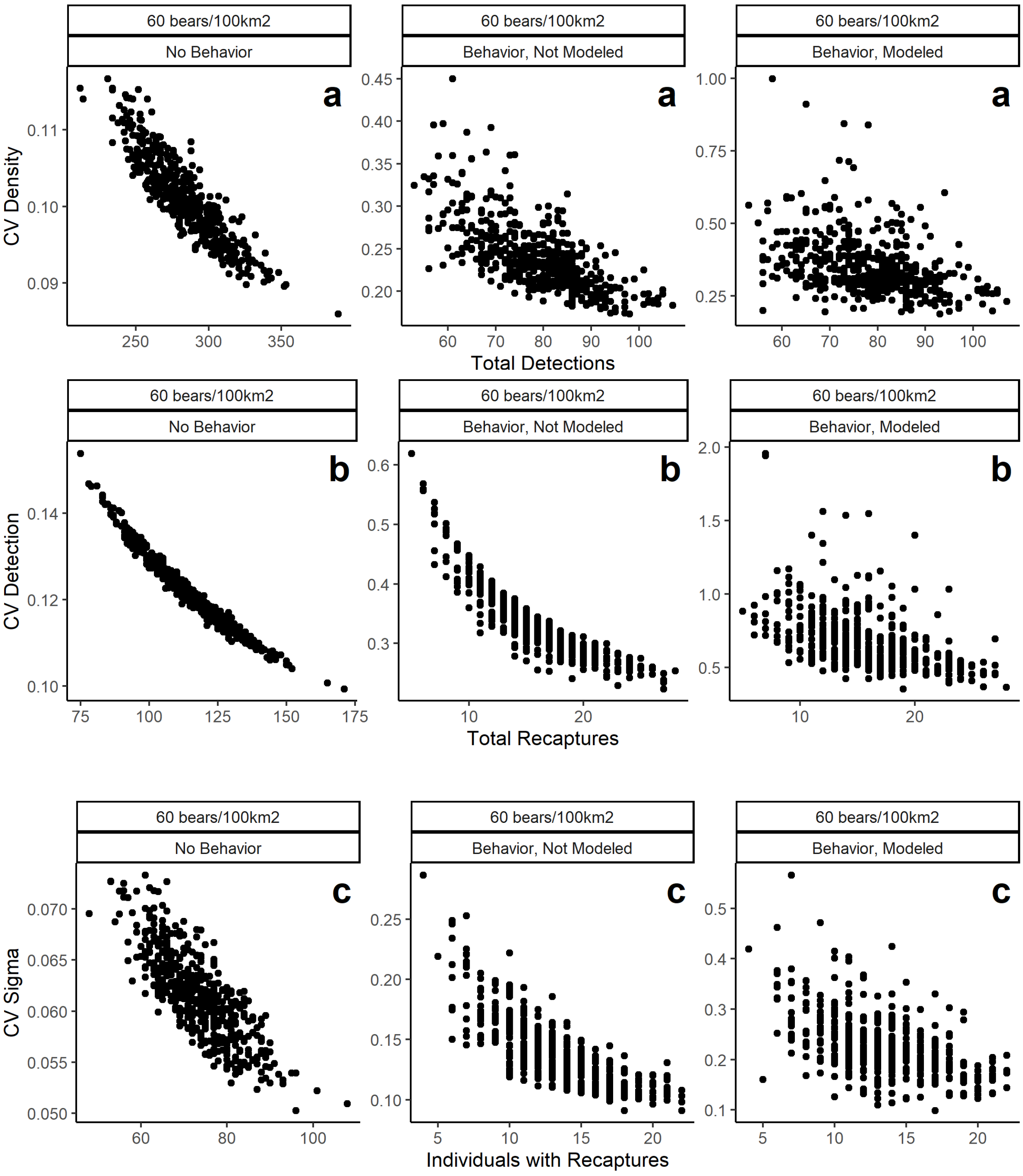


*Figure S4. The relationship between SCR parameter estimates of a) density, b) detection and c) sigma and the best-supported sampling data attribute from analysis of the empirical datasets*
