# Supplementary material for: Precision and bias of spatial capture–recapture estimates: A multi‐site, multi‐year Utah black bear case study
Source: Ecol Appl. 2022 May 17;32(5):e2618. doi: 10.1002/eap.2618 (PMC9287071; doi:10.1002/eap.2618)
Supplement: Supplementary file 3 — Appendix S3 [file EAP-32-0-s003.pdf]

Precision and bias of spatial capture–recapture estimates: A multi-site, multi-year Utah black bear case study

Greta M. Schmidt, Tabitha A. Graves, Jordan C. Pederson, Sarah L. Carroll

Ecological Applications

Appendix S3: Visualizing the relationships of standard error and coefficient of variation for estimates of density, detection, and sigma with the sampling data characteristics hypothesized to influence precision.

Section S1: Summary

Visualizing relationships of standard error and coefficient of variation for estimates of density, detection, and sigma with the sampling data characteristics hypothesized to influence precision. In each figure, graphs are ordered L-R, top to bottom row in order of support (AIC-based model selection) of each associated model.

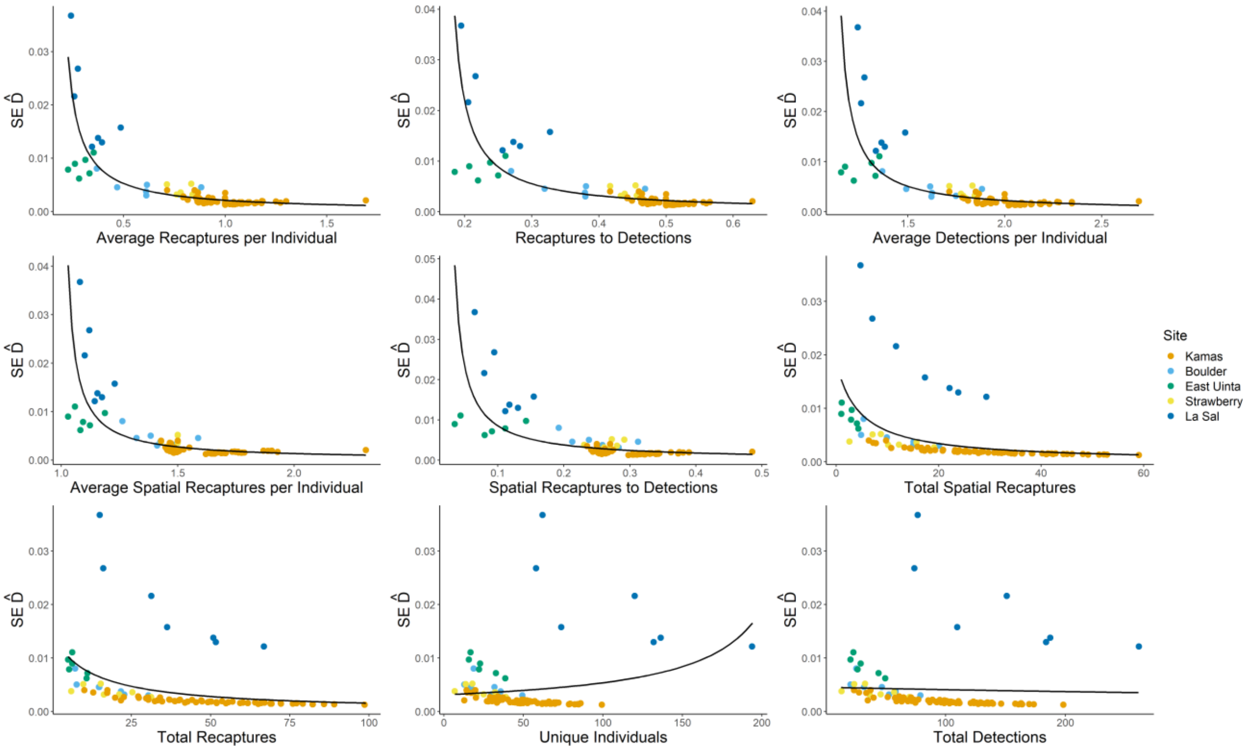

Figure S1. Standard error of the density estimates versus summarized attributes of the sampling data hypothesized to influence precision

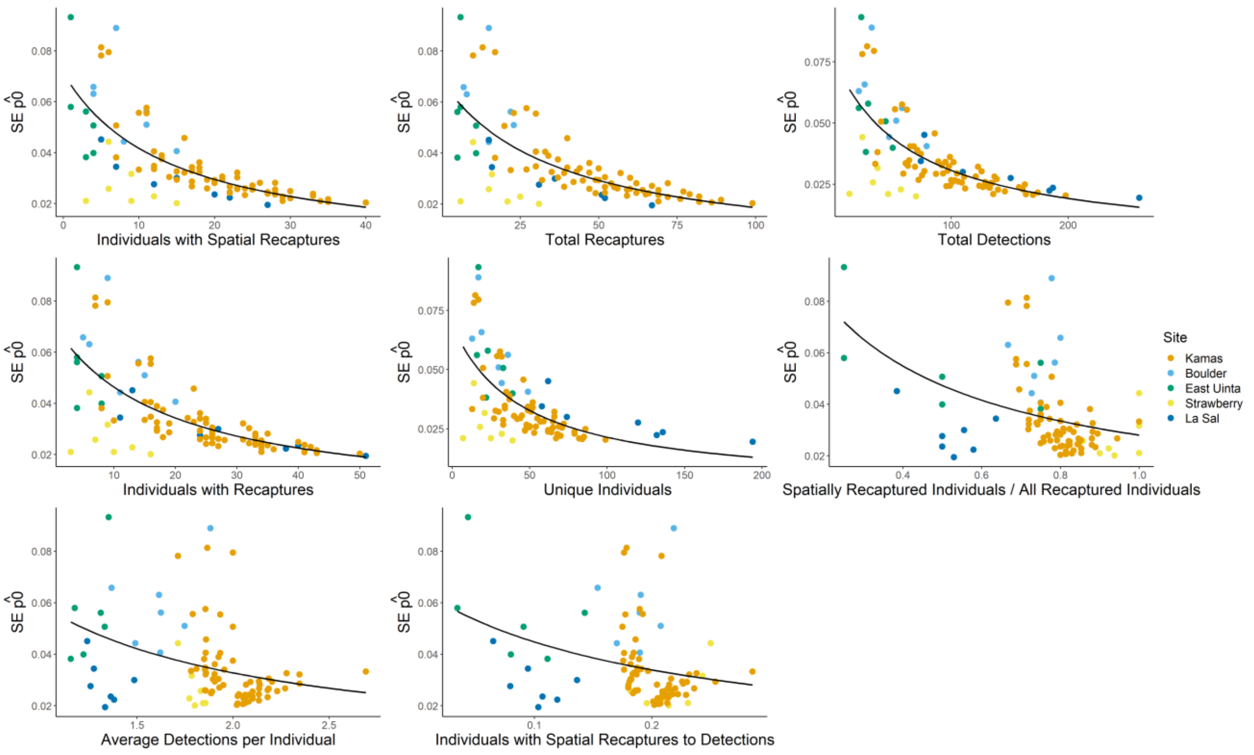

Figure S2. Standard error of the detection estimates versus summarized attributes of the sampling data hypothesized to influence precisions

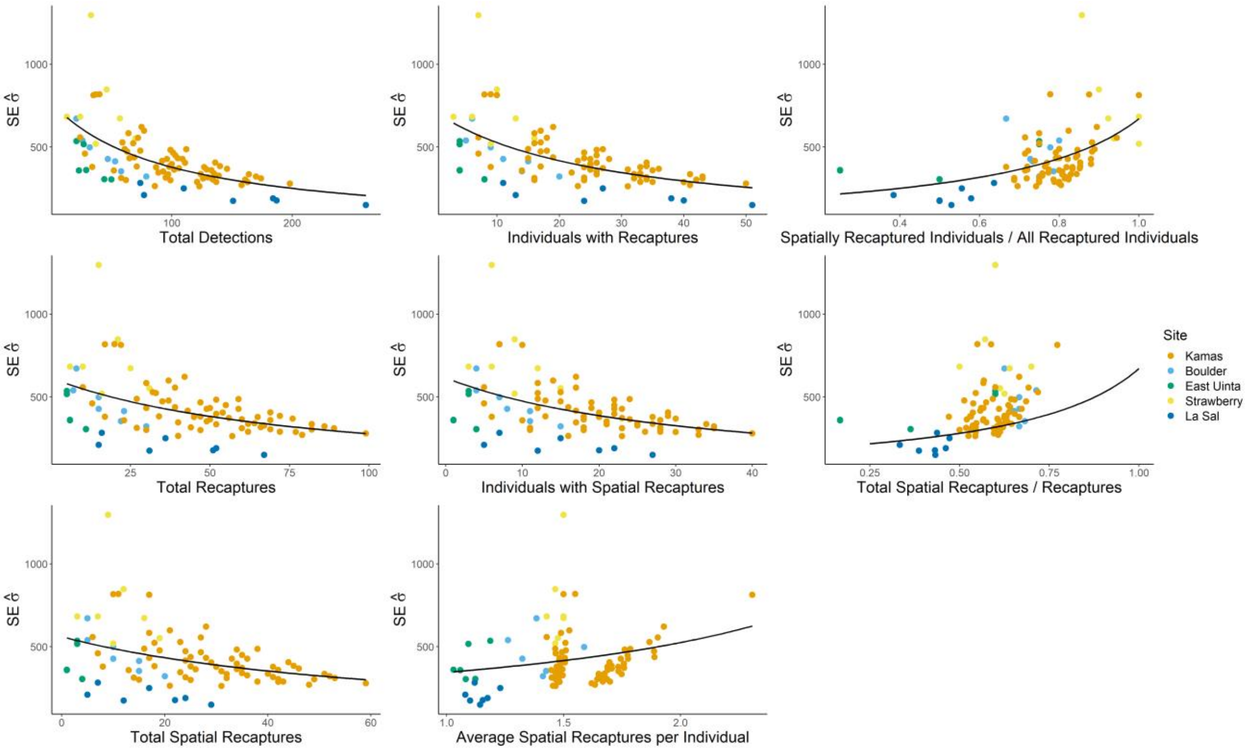

Figure S3. Standard error of the sigma estimates versus summarized attributes of the sampling data hypothesized to influence precisions

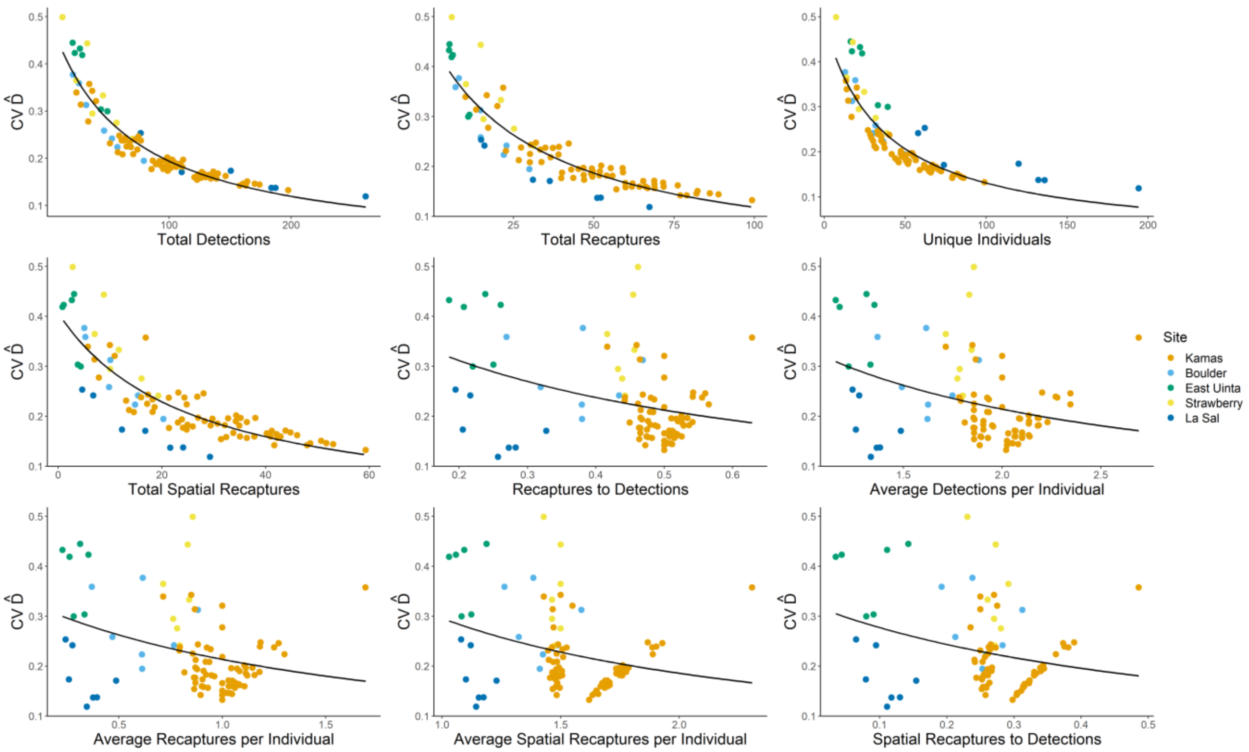

Figure S4. Coefficient of variation of the density estimates versus summarized attributes of the sampling data hypothesized to influence precision

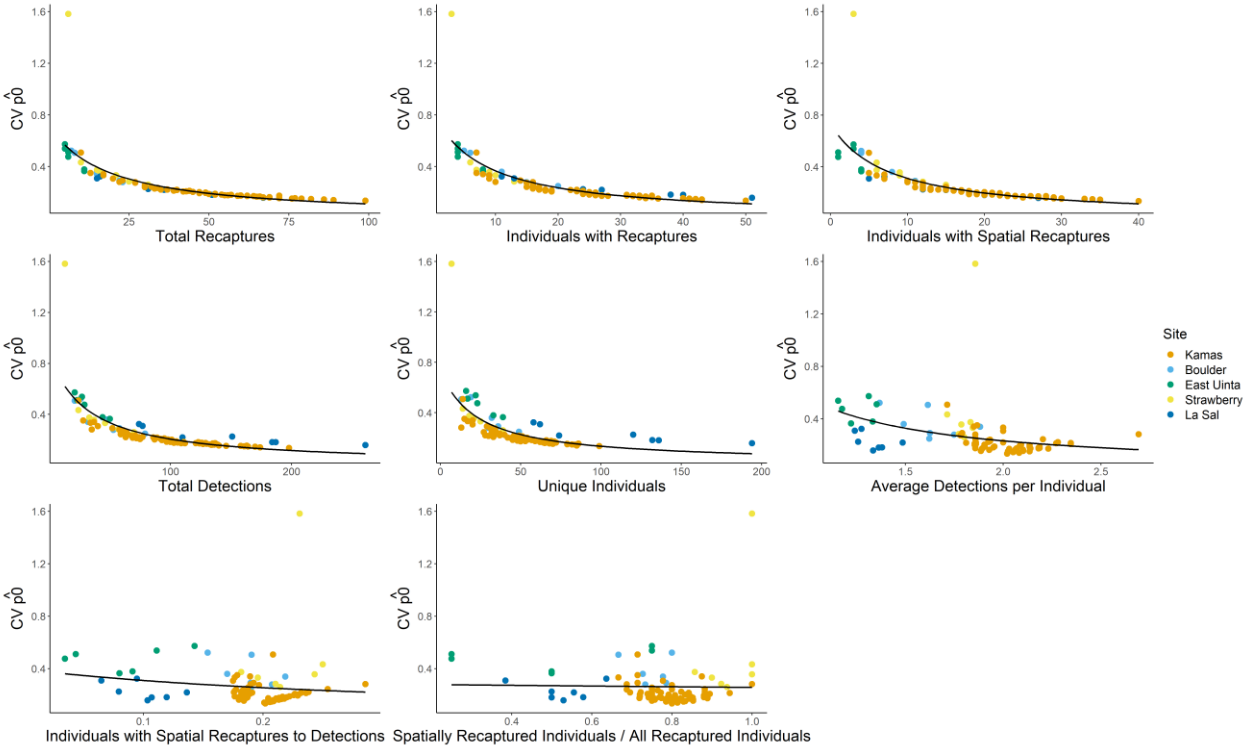

Figure S5. Coefficient of variation of the detection estimates versus summarized attributes of the sampling data hypothesized to influence precision

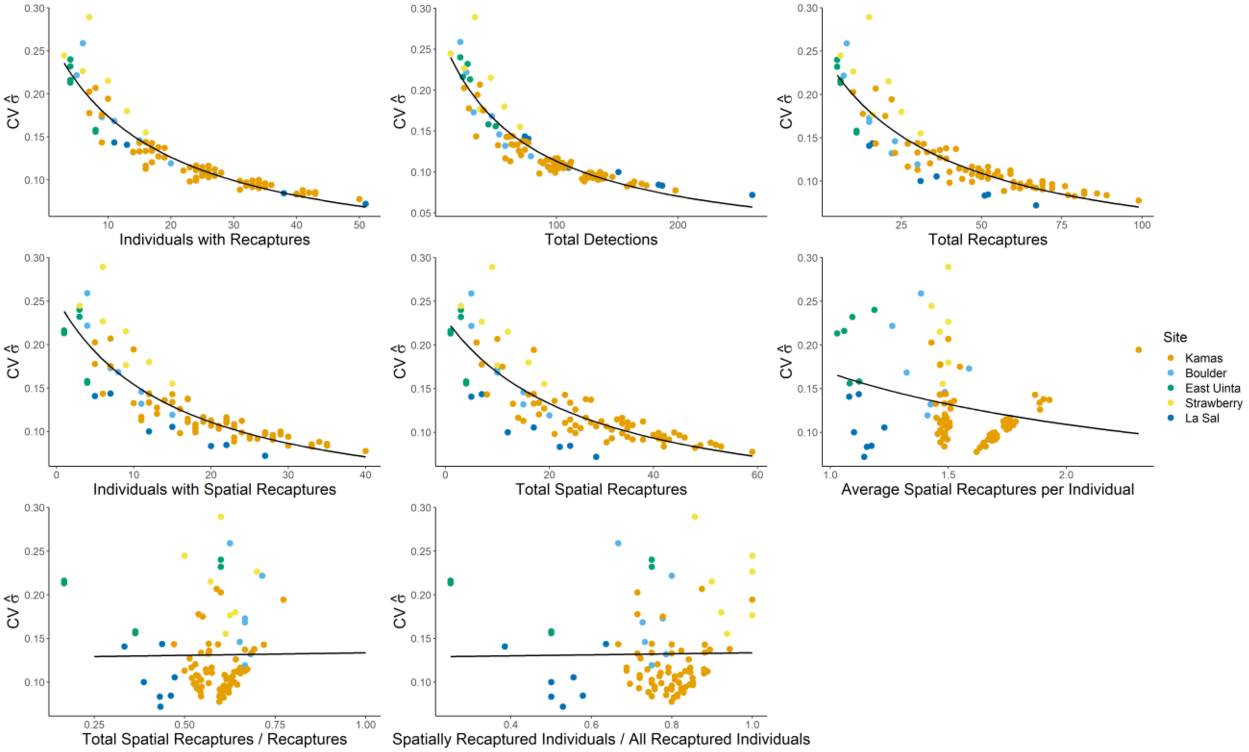

Figure S6. Coefficient of variation of the sigma estimates versus summarized attributes of the sampling data hypothesized to influence precision
